# *Lacticaseibacillus paracasei* JY062 Postbiotic Alleviated 3% DSS-Induced Colitis in Mice via Integrated Antioxidant, Barrier Repair, Immunomodulatory and Microbiota Modulation

**DOI:** 10.3390/antiox14101256

**Published:** 2025-10-19

**Authors:** Jinfeng Guo, Yilin Sun, Kaiqi Gao, Haijie Zhao, Yue Su, Ying Zhao, Yu Zhang, Yujun Jiang

**Affiliations:** 1 Key Laboratory of Dairy Science, Ministry of Education, College of Food Science, Northeast Agricultural University, Harbin 150031, China; b211001001@neau.edu.cn (J.G.); b221001010@neau.edu.cn (Y.S.); s241001006@neau.edu.cn (K.G.); s231001085@neau.edu.cn (H.Z.); h10092@neau.edu.cn (Y.S.); s221002168@neau.edu.cn (Y.Z.); 2 Food Laboratory of Zhongyuan, Luohe 462300, China

**Keywords:** postbiotics, antioxidant, ulcerative colitis, intestinal barrier, intestinal inflammation, gut homeostasis

## Abstract

Ulcerative colitis (UC) with multifactorial etiology remains clinically challenging. While current pharmacotherapies alleviate symptoms, their long-term application is constrained by significant side effects. *Lacticaseibacillus paracasei* JY062 postbiotic (Pa JY062) exerts multi-target therapeutic effects via integrated antioxidant, barrier repair, immunomodulatory and microbiota modulation for UC. Pa JY062 exhibited potent antioxidant capacity and reduced reactive oxygen species (ROS) in Caco-2 cells. In DSS-colitis mice, high-dose Pa JY062 (HP, 800 mg/kg) restored intestinal barrier integrity (Evans blue permeability: 0.0547 vs. 0.107, *p* < 0.01), enhanced antioxidant defenses (SOD: 9.43 vs. 5.5; GSH-Px: 62.74 vs. 40.94 U/mg protein, *p* < 0.01), and rebalanced Th1/Th2/Th17/Treg responses. *LigiLactobacillus murinus* (*L. murinus*) was enriched, while *Bacteroides acidifaciens* (*B. acidifaciens*) was diminished, SCFAs increased (acetate 2772.70 ± 21.08, propionate 669.84 ± 6.79, butyrate 324.14 ± 0.42 μg/g). Spearman correlations linked *L. murinus* to barrier proteins (ZO-1/Occludin/E-cadherin), contrasting *B. acidifaciens*–inflammation associations. These findings revealed that Pa JY062 exerted multi-target therapeutic effects on UC, and offered a wider array of options for intestinal health. Pa JY062 represents a promising natural antioxidant-based strategy for UC management.

## 1. Introduction

UC is a complex form of inflammatory bowel disease (IBD) that emerges from the intricate interaction of genetic susceptibility, environmental factors, disturbances in the intestinal microbiota, and immune system dysfunction [1]. This multifactorial pathogenesis challenges monotherapies targeting isolated pathways. Current standard-of-care treatments, including 5-aminosalicylic acid (5-ASA) for mucosal anti-inflammation, glucocorticoids for acute flare control, and anti-TNFα monoclonal antibodies for systemic immune modulation, effectively suppress acute inflammation but fail to address upstream drivers, leading to high relapse rates and adverse effects (e.g., osteoporosis, opportunistic infections) [2]. Current medications with singular regulatory effects are insufficient to achieve complete resolution of UC, highlighting the need for more comprehensive therapeutic strategies. The restoration of intestinal barrier integrity, increase in intestinal microbial diversity and attenuation of inflammatory responses are fundamental to UC remission. However, targeting the regulation of oxidative stress is also a crucial intervention strategy in the pathogenesis of UC. Research has shown that a nanoparticle targeting the ROS-responsive achieved a synergistic effect in IBD therapy [3].

Consuming an antioxidant-rich diet not only fulfills daily nutritional requirements but also alleviates inflammatory responses. Furthermore, emerging evidence has demonstrated its efficacy in modulating gut homeostasis represents a promising adjunctive strategy, especially for postbiotics [4]. Postbiotic defined as a “preparation of inanimate microorganisms and/or their components that confers a health benefit on the host” [5], postbiotic has gained traction as dietary modulators of gut homeostasis. Postbiotics from *Lactobacillus plantarum* JM015 [6], extracellular vesicles derived from *Bifidobacterium longum* NSP001 [7] and montmorillonite composite postbiotics [8] have shown effectiveness in alleviating intestinal inflammation and enhancing gut health. However, while current postbiotic interventions primarily target inflammatory resolution, barrier restoration, and microbiota modulation, their direct impact on oxidative stress remains largely unexplored. This gap in the knowledge highlights the potential of postbiotics with integrated antioxidant properties.

In our previous work, we discovered that *Lacticaseibacillus paracasei* JY062 can alleviate colitis in mice, and its postbiotic Pa JY062, formulated as a food-grade skimmed-milk-based preparation, possessed potent anti-inflammatory properties and the reestablished the intestinal barrier [9,10]. Therefore, this study aims to comprehensively evaluate the therapeutic potential of Pa JY062 against colitis, with a focus on its antioxidant capacity. We assessed its ability to scavenge ROS in Caco-2 cells in vitro and investigated its effects in a DSS-induced colitis mouse model. The investigation encompassed systemic antioxidant enzymes (SOD, GSH-Px), gut barrier proteins (ZO-1, occludin), immune cytokines (IFN-γ, IL-17A), and microbiota–short-chain fatty acid (SCFA) interactions.

## 2. Materials and Methods

### 2.1. Analysis of Antioxidant Capacity

A *Lacticaseibacillus rhamnosus* GG (LGG) postbiotic was used as a positive control, while Trolox, a water-soluble vitamin E analog, served as the antioxidant standard. The blank was the negative control. The preparation process of postbiotic LGG was the same as that of Postbiotic Pa JY062 [9].

The DPPH radical scavenging activity was assessed utilizing DPPH radical scavenging ability Kit (Nanjing Jiancheng Bioengineering Institute, Nanjing, China). Postbiotics were homogenized in 80% methanol solution at 1:10 (*m*/*v*), and centrifuged (12,000 rpm, 10 min, 4 °C) to obtain the supernatant. The remaining procedures, conducted at 4 °C unless otherwise specified, are detailed in Table A1. Briefly, the mixture was incubated at 25 °C for 30 min in the dark and then centrifuged at 4000 rpm for 5 min. OD_517nm_ was measured by a microplate reader (BioTek, WA, USA).

DPPH free radical scavenging rate (%) = (1 – (*A*_determination_ – *A*_control_))/(*A*_blank_) × 100

Total antioxidant capacity (T-AOC) was quantified utilizing a T-AOC Assay Kit (Nanjing Jiancheng Bioengineering Institute, Nanjing, China). Postbiotics were homogenized in sterile water at a 1:9 (*w*/*v*) ratio and vortex-mixed for complete antioxidant extraction. Following centrifugation (12,000× *g*, 5 min, 4 °C), supernatants were meticulously collected for subsequent research. The remaining operations were performed according to the Table A2. Reaction mixtures were incubated at 25 °C for 6 min in 96-well plates, after which OD_405nm_ was measured using a microplate reader (BioTek, Shoreline, WA, USA).

### 2.2. Cell Culture

Caco-2 cells were obtained from the Key Laboratory of Dairy Science at the Ministry of Education, Northeast Agricultural University, Harbin, China. All cells, between passages 5 and 20, were maintained in high-glucose DMEM supplemented with 10% fetal bovine serum and 1% penicillin–streptomycin, then maintained at 37 °C in a 5% CO_2_-humidified incubator.

### 2.3. Reactive Oxygen Species Assay

Caco-2 cells were seeded at a density of 1 × 10^4^ cells per well were plated in 96 well plates and treated with LPS (10 μg/mL) for 12 h, followed by Pa JY062 (250 μg/mL) with/without exposure for 24 h. ROS levels were assayed by ROS kit (Beyotime, Shanghai, China). Cells were incubated in DMEM containing DCFH-DA (10 μM) in the dark at 37 °C for 30 min. The ROS images was recorded by the EVOS FL Auto Cell Imaging System (Thermo Fisher Scientific, MA, USA). Unstimulated Caco-2 cells served as negative controls, with three replicates established for each treatment group.

### 2.4. Analysis of Bioactive Components in Pa JY062

The comprehensive metabolites of postbiotics Pa JY062 was detected using the T500 targeted platform (MetWare, http://www.metware.cn/ (accessed on 15 March 2024)) equipped with AB Sciex QTRAP 6500 LC-MS/MS system, as detailed in our previous publication [9]. This approach identified 198 newly detected components in Pa JY062 postbiotic. A literature search was conducted to identify metabolites with reported bioactivities, including antioxidant, anti-inflammatory, intestinal barrier-repairing, and gut microbiota-modulating properties.

### 2.5. Animal Experimental Design

The animal experimentation protocol received approval from the Animal Ethics Committee of Northeast Agricultural University to ensure the utmost adherence to animal welfare, with the experiment designated as NEAUEC20230422, approval on 9 November, 2023. Sixty 8-week-old male BALB/c mice (weight: 20 ± 2 g) were acquired from Chengdu Dossy Experimental Animals Co., Ltd. (Chengdu, China). All mice were randomly divided into 6 groups (*n* = 10): control group (Ctrl, sterile water), DSS group (DSS, 3% DSS solution), 5-aminosalicylic acid group (ASA, 200 mg/kg), low-dose Pa JY062 (LP, 3.0% DSS + 200 mg/kg Pa JY062), medium-dose Pa JY062 (MP, 3.0% DSS + 500 mg/kg Pa JY062), and high-dose Pa JY062 (HP, 3.0% DSS + 800 mg/kg Pa JY062).

Mice were acclimatized for 7 days under standard conditions: temperature of 23 ± 2 °C, relative humidity of 55 ± 10%, and a 12 h light/dark cycle. Then, all groups, except for the Ctrl group, were granted unrestricted access to 3% DSS to establish a model of colitis (day 0 to day 7). During the remission period (day 8 to day 14), Ctrl and DSS groups received 200 μL of sterile water daily. LP, MP, and HP groups were given daily 200 μL of Pa JY062 solutions. The ASA group was gavaged daily with 200 μL of a 5-ASA solution. The daily food intake, water intake, and body weight changes in all mice were recorded. Fecal samples from each mouse were collected daily into a 1.5 mL sterile centrifuge tube. On day 15, all mice were humanely euthanized under intraperitoneal anesthesia with 10% chloral hydrate. The length of the colon was assessed, and the spleen and thymus were subjected to weighing. Three mice from each group were selected to assess Evans blue colonic permeability. One segment of the fresh colon was frozen at −80 °C, another segment was preserved in 2.5% glutaraldehyde, and the final segment was fixed in 4% paraformaldehyde for subsequent analyses. The spleen, thymus, serum, and cecal contents were harvested and preserved at −80 °C.

### 2.6. Disease Activity Index

Disease activity index (DAI) was computed according to the methodology outlined by Zhu et al. [11].

DAI = (*weight loss score* + *stool consistency score* + *bleeding score*)/3

### 2.7. Evans Blue Permeability and Ultrastructural Analysis

The colonic permeability of the mice was evaluated employing the Evans blue method as delineated by Rizvi [12].

For ultrastructural analysis, the distal colon was sectioned into 1 mm^2^ sections along the intestinal axis and subsequently preserved in precooled 2.5% glutaraldehyde at 4 °C for 12 h. Sample pieces were rinsed 3 times with 0.1 M PBS (15 min/time), stabilized with 1% OsO4 for 30 min, and rinsed again with PBS buffer 3 times. Dehydration was carried using ethanol series. The subsequent procedures followed the procedure detailed by Wang [13].

### 2.8. Histopathological Staining

A 1 cm^2^ piece of fresh distal colon was immersed in 4% neutral formaldehyde solution for 24 h. The tissue edges were then neatly trimmed, subjected to gradient dehydration in a dehydrator, and finally immersed in wax for embedding. The wax block was sliced into 4 μm sections, dewaxed in xylene, and rehydrated using a series of ethanol solutions. Colonic tissues were subjected to staining with hematoxylin and eosin (H&E) to evaluate colonic injury [14]. To identify goblet cells, Alcian blue periodic acid–Schiff (AB-PAS) staining was conducted employing the AB-PAS Stain Kit (Solarbio, Beijing, China), following the instructions provided by the manufacturer. The immunohistochemistry (IHC) procedure was carried out following the protocol established in [15].

### 2.9. Real-Time Quantitative PCR

The colon tissue was cut into 5 mm^2^ pieces and ground in liquid nitrogen. One part was treated with RNAplus to extract total RNA, another was treated by RIPA lysis buffer (Biosharp, Hefei, China) and preserved at −80 °C for later enzyme-linked immunosorbent assay.

RNA concentration was measured by Nanodrop (Waltham, MA, USA) and recorded in Table A3. The RNA was transcribed into cDNA, and a real-time quantitative polymerase chain reaction (RT-qPCR) was employed to ascertain the transcript levels of colonic genes utilizing the 2^−ΔΔCt^ method. GAPDH served as the housekeeping gene. The primers employed for the RT-qPCR examination are delineated in Table A4.

### 2.10. Evaluation of Oxidative Stress

The glutathione peroxidase (GSH-PX) test kit (Nanjing Jiancheng Bioengineering Institute, Nanjing, China), and total superoxide dismutase (T-SOD) assay kit (Nanjing Jiancheng Bioengineering Institute, Nanjing, China) were utilized to quantify the levels of GSH-px and T-SOD in the mouse colon. The comprehensive procedural steps are delineated in Appendix A.1.

### 2.11. Enzyme-Linked Immunosorbent Assay

Colon tissue lysate was centrifuged at 3000× *g* for 5 min to harvest the supernatant for the assessment of cytokine (IL-2, IL-4, IL-10, IFN-γ) and cell surface adhesion molecules (VCAM-1, ICAM-1) concentrations, in accordance with the manufacturer’s instructions (Xinle, Shanghai, China).

### 2.12. Gut Microbiota Analysis

Genomic DNA from samples was extracted using the CTAB method. The V3-V4 region of the gut microbiota’s 16S rRNA gene was amplified via PCR. The 1× loading buffer (containing SYBR Green) was combined with the PCR products, which were then subjected to electrophoresis on a 2% agarose gel for visualization. The PCR products were mixed in equimolar ratios. This mixture was purified using the Qiagen Gel Extraction Kit (Qiagen, Germany). Sequencing libraries were prepared in accordance with the manufacturer’s guidelines with the TruSeq^®^ DNA PCR-Free Sample Preparation Kit (Illumina, CA, USA), and index codes were incorporated. The quality of the libraries was evaluated using the Qubit@ 2.0 Fluorometer (Thermo Scientific, MA, USA) and the Agilent Bioanalyzer 2100 system. Sequencing was conducted using the Illumina NovaSeq system, generating 250 bp paired-end reads. For details on 16S rRNA analysis, see Appendix A.2.

### 2.13. Detection of Short-Chain Fatty Acids

SCFAs levels were analyzed by MetWare (http://www.metware.cn/ (accessed on 15 March 2024)) utilizing the Agilent 8890-7000D GC-MS/MS system. For detailed operation steps, see Appendix A.3.

### 2.14. Statistical Analysis

Statistical analyses were carried out with IBM SPSS Statistics 25.0. For data exhibiting a normal distribution (mean ± SEM), comparisons were made via one-way ANOVA, followed by Duncan’s post hoc test. For microbial abundance (phylum/genus/species), nonparametric Kruskal–Wallis tests were applied. Spearman’s correlation assessed associations.

## 3. Results

### 3.1. Antioxidant Capacity of Pa JY062 Postbiotics

**Figure 1:**
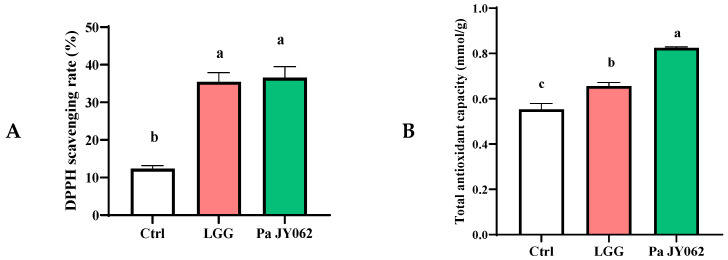
The antioxidant capacity of postbiotic Pa JY062 (*n* = 3 biological replicates). (**A**) The DPPH scavenging rate (%) of postbiotic Pa JY062. (**B**) The total antioxidant capacity of postbiotic Pa JY062. The different letters indicate statistically significant differences (*p* < 0.05).

The antioxidant capacity of Pa JY062 was systematically evaluated through DPPH radical scavenging and T-AOC assays. As shown in Figure 1A, the DPPH inhibition rate of Pa JY062 (36.54 ± 2.9%) was 2.95-fold higher than that of the blank control (Ctrl, 12.36 ± 0.83%; *p* < 0.01) and statistically indistinguishable from the LGG postbiotic positive control (35.46 ± 2.4%). The potent antioxidant activity of Pa JY062 was further confirmed by the T-AOC assay (Figure 1B). The T-AOC value of Pa JY062 (0.82 ± 0.01 mmol Trolox equiv/g) was significantly higher (*p* < 0.05) than both the LGG postbiotic (0.65 ± 0.01 mmol/g) and the blank control (0.55 ± 0.02 mmol/g).

### 3.2. The Influence of Pa JY062 on Reactive Oxygen Species

**Figure 2:**
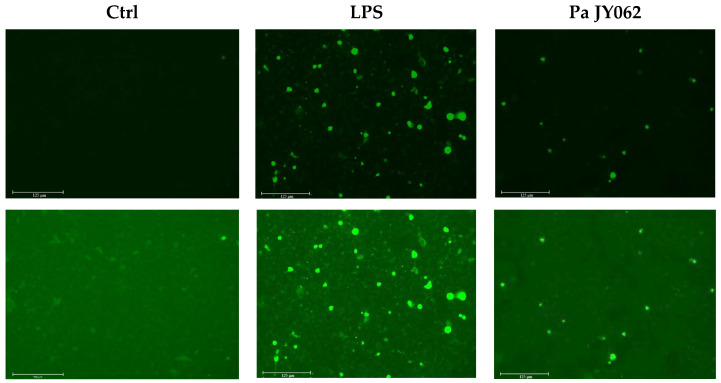
The effect of PaJY062 on ROS production in Caco-2 cell (*n* = 3 biological replicates). DCFH-labeled Caco-2 cell fluorescence microscopy treated for 24 h by Pa JY062 (250 μg/mL).

To investigate the protective effect of Pa JY062 against oxidative stress, intracellular ROS levels in Caco-2 cells were assessed by fluorescence imaging. The LPS resulted in a significant increase in green fluorescence in Caco-2 cells compared to the Ctrl. Pa JY062 markedly reduced the green fluorescence relative to LPS (Figure 2, *p* < 0.001).

### 3.3. Bioactive Ingredients of Pa JY062

**Figure 3:**
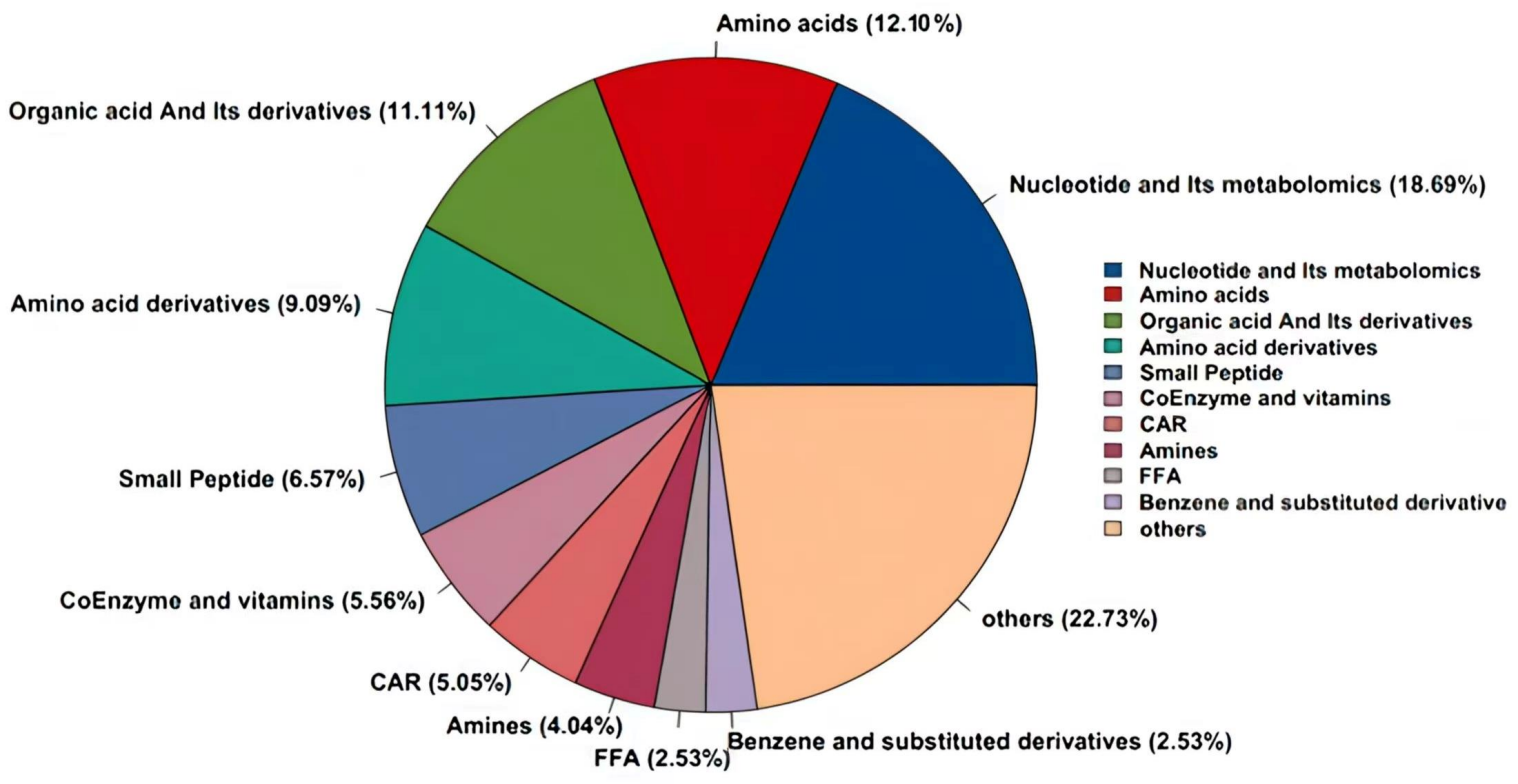
The pie chart of Pa JY062 postbiotic components classification and the percentage of each substance.

T500-targeted metabolomic profiling of Pa JY062 identified 198 metabolites (Table 1). Compositional analysis revealed that the profile was quantitatively dominated by five major five classes. Nucleotides and their metabolites (18.69%), amino acids (12.12%), organic acids and derivatives (11.11%), amino acid derivatives (9.09%), and small peptides (6.57%) (Figure 3). Based on the established literature, 31 of these metabolites were annotated with putative functions falling into four primary categories: antioxidant activity, anti-inflammatory effects, intestinal barrier repair, and gut microbiota modulation. Notable functional metabolites included amino acids (e.g., histidine) and organic acid derivatives (e.g., allantoin, citric acid, 3-phenyllactic acid). Together, these two classes accounted for 42% of all annotated functional metabolites (Table A5).

### 3.4. Pa JY062 Mitigated DSS-Induced Colitis Progression

**Figure 4:**
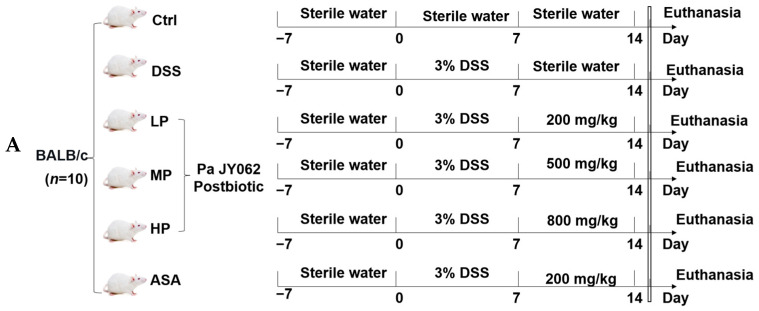

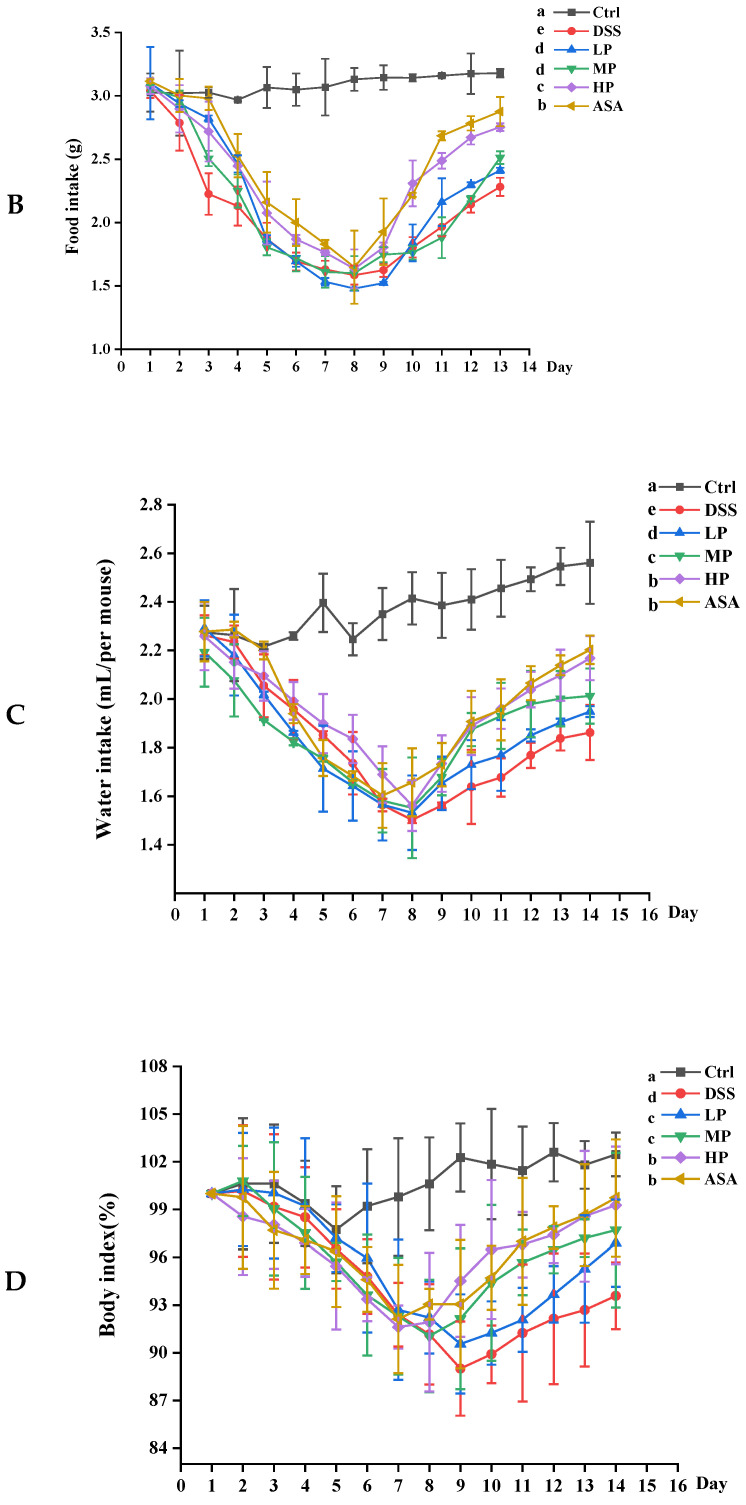

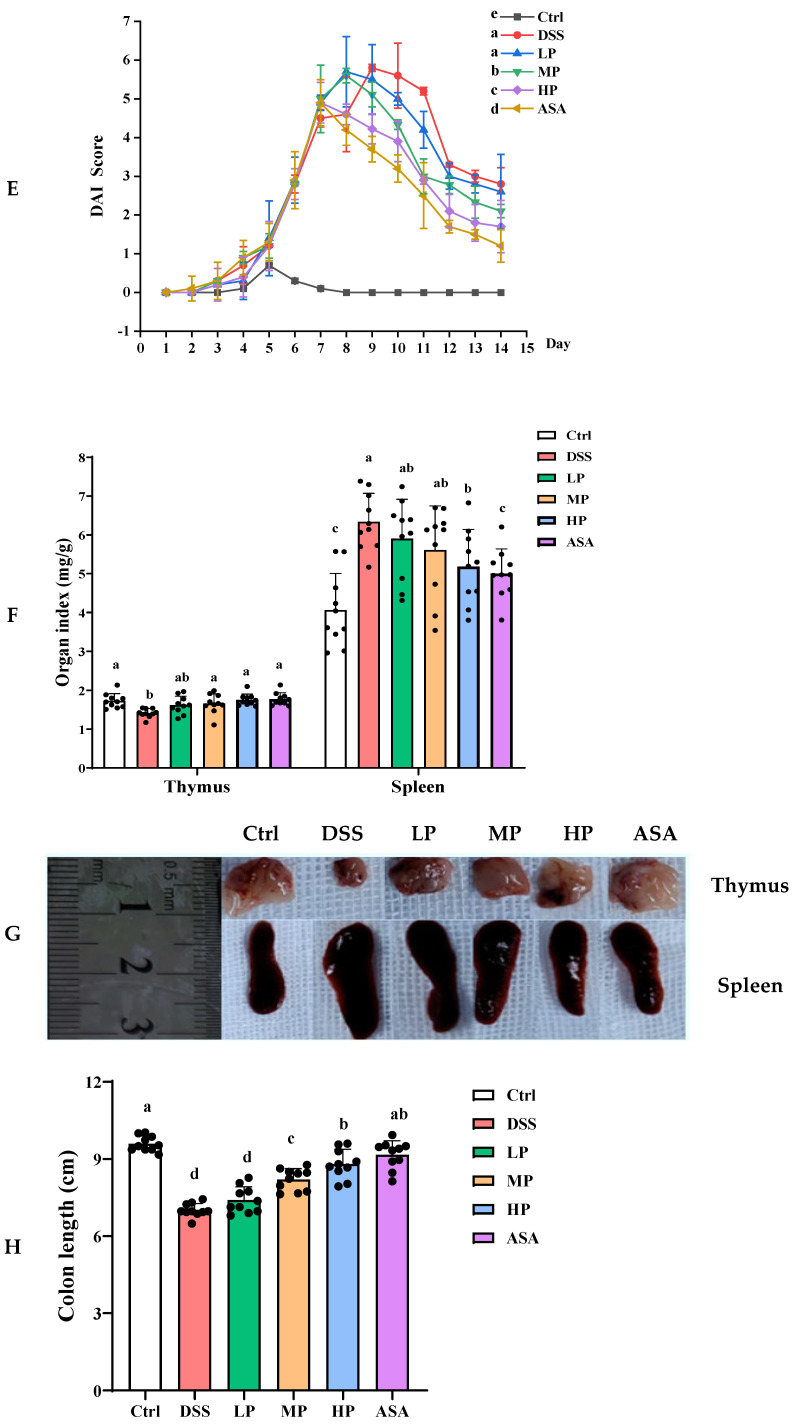

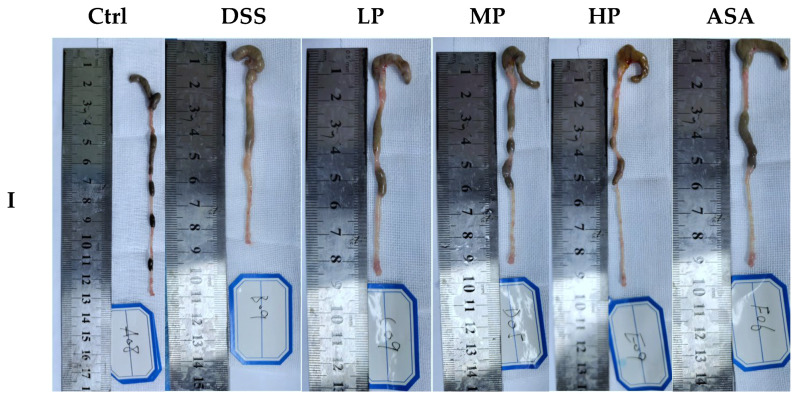
Efficacy of postbiotic Pa JY062 on 3% dextran sodium sulfate (DSS)-induced acute colitis (*n* = 10). (**A**) Experimental protocol design. (**B**) Food intake. (**C**) Water intake. (**D**) Body index. (**E**) Disease activity index (DAI). (**F**) Organ index. (**G**) Physical picture in the thymus and spleen. (**H**) Colon length. (**I**) Physical picture of colon length. The different letters indicate statistically significant differences (*p* < 0.05).

Pa JY062 significantly mitigated the progression of DSS-induced colitis, as schematically outlined in the experimental timeline (Figure 4A). The administration of DSS successfully induced colitis, as evidenced by progressive clinical manifestations such as significant body weight loss, elevated disease activity index (DAI), and reductions in food and water intake (Figure 4B–E). Following DSS withdrawal (Day 8), mice treated with Pa JY062, particularly at a high dose (HP), exhibited a significantly accelerated recovery in these clinical parameters compared to the DSS group. The HP group demonstrated comparable therapeutic efficacy to the positive control ASA (Figure 4B–E, Day 13, *p* < 0.001). Furthermore, Pa JY062 attenuated DSS-induced immune organ pathology, ameliorating splenomegaly (spleen index: HP 5.18 ± 95 mg/g vs. DSS 6.34 ± 0.73 mg/g, *p* < 0.01) and thymic atrophy (thymus index: HP 1.75 ± 0.15 mg/g vs. DSS 1.41 ± 0.11 mg/g, *p* < 0.01; Figure 4F,G). The HP group also exhibited significant prevention of DSS-induced colon shortening (colon length: HP 8.80 ± 0.57 cm vs. DSS 7.01 ± 0.26 cm, *p* < 0.01; Figure 4H,I). Hence, Pa JY062 mitigated 3% DSS-induced colitis progression.

### 3.5. Pa JY062 Mitigated Damage to the Colonic Barrier in DSS Mice

**Figure 5:**
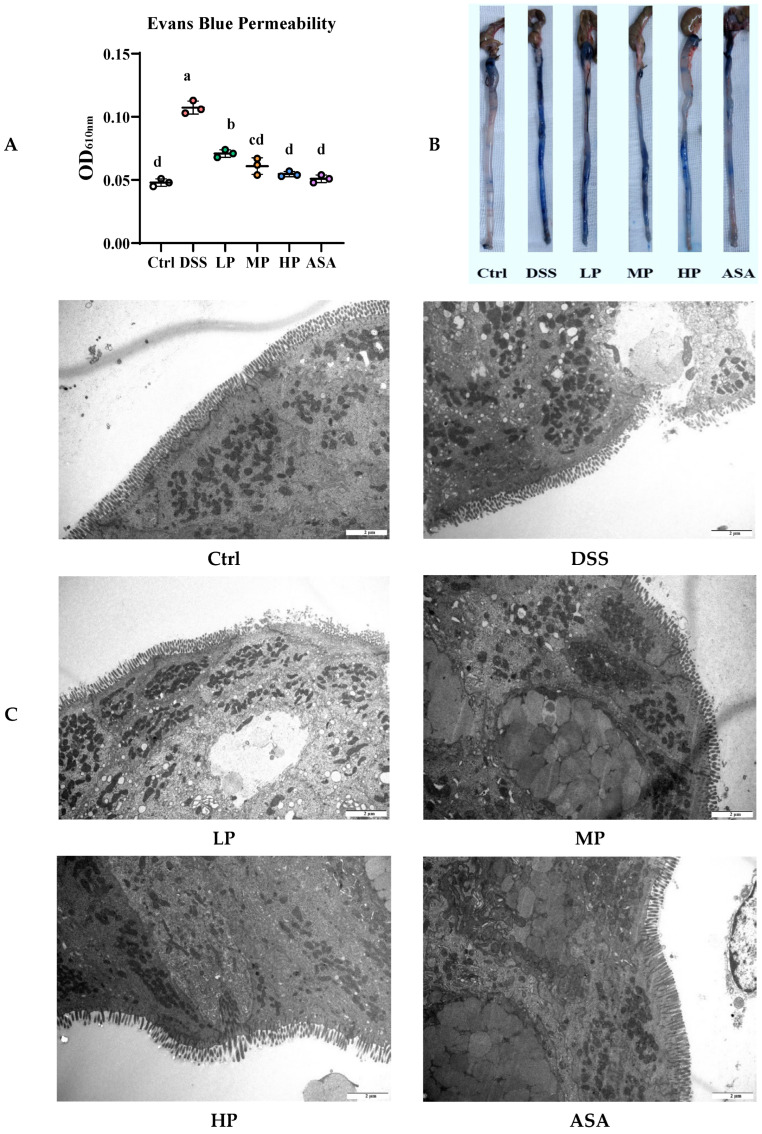

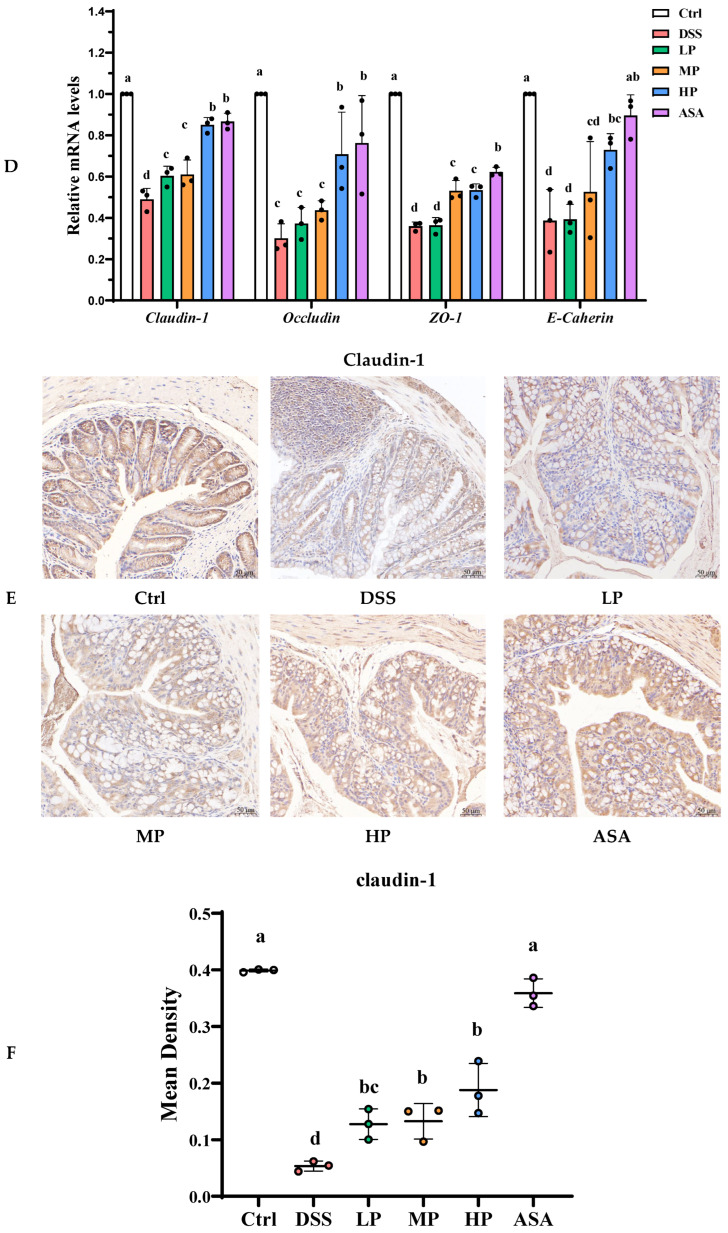

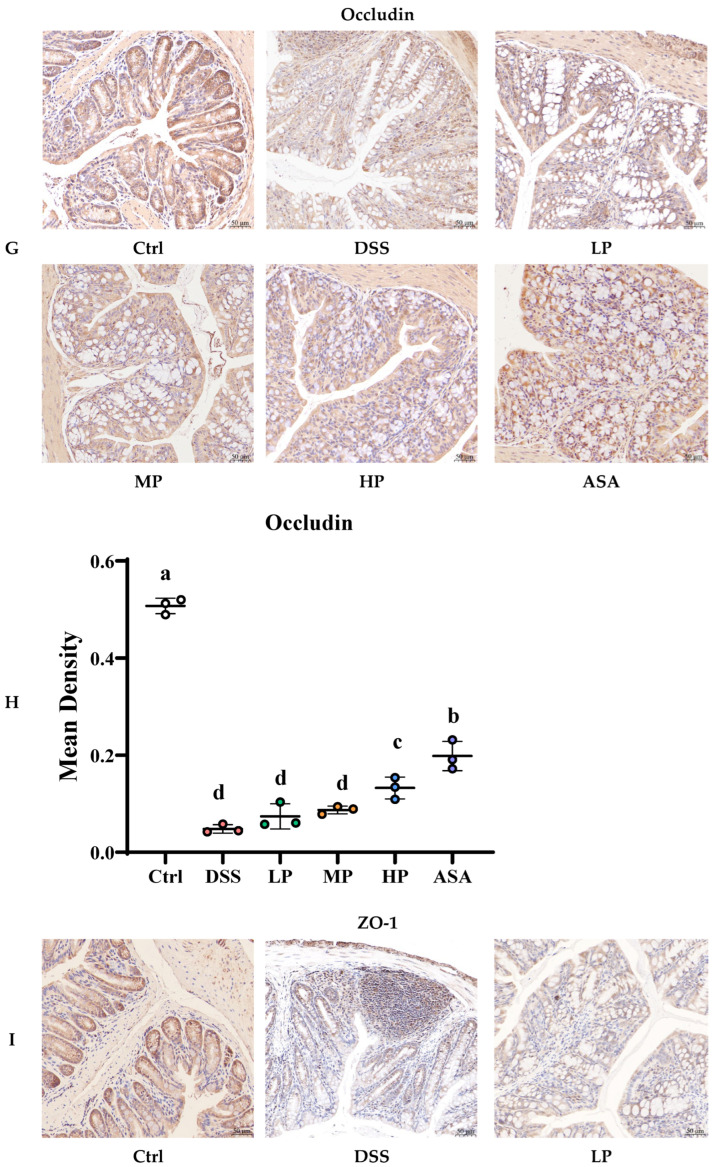

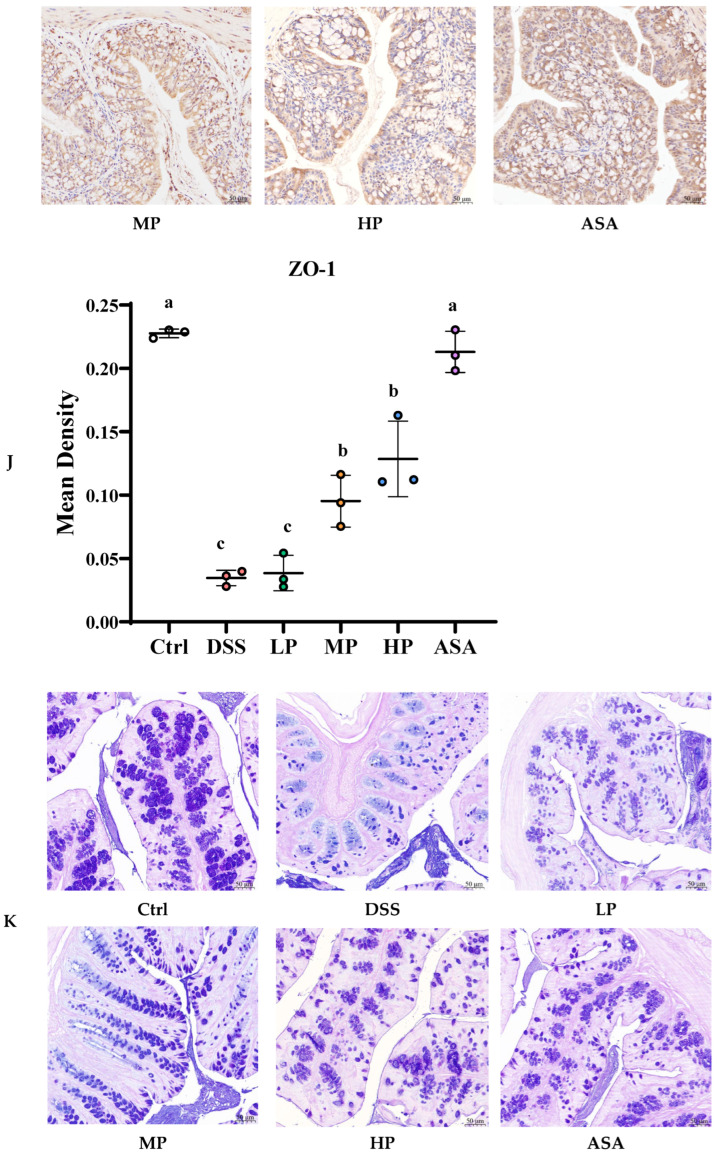

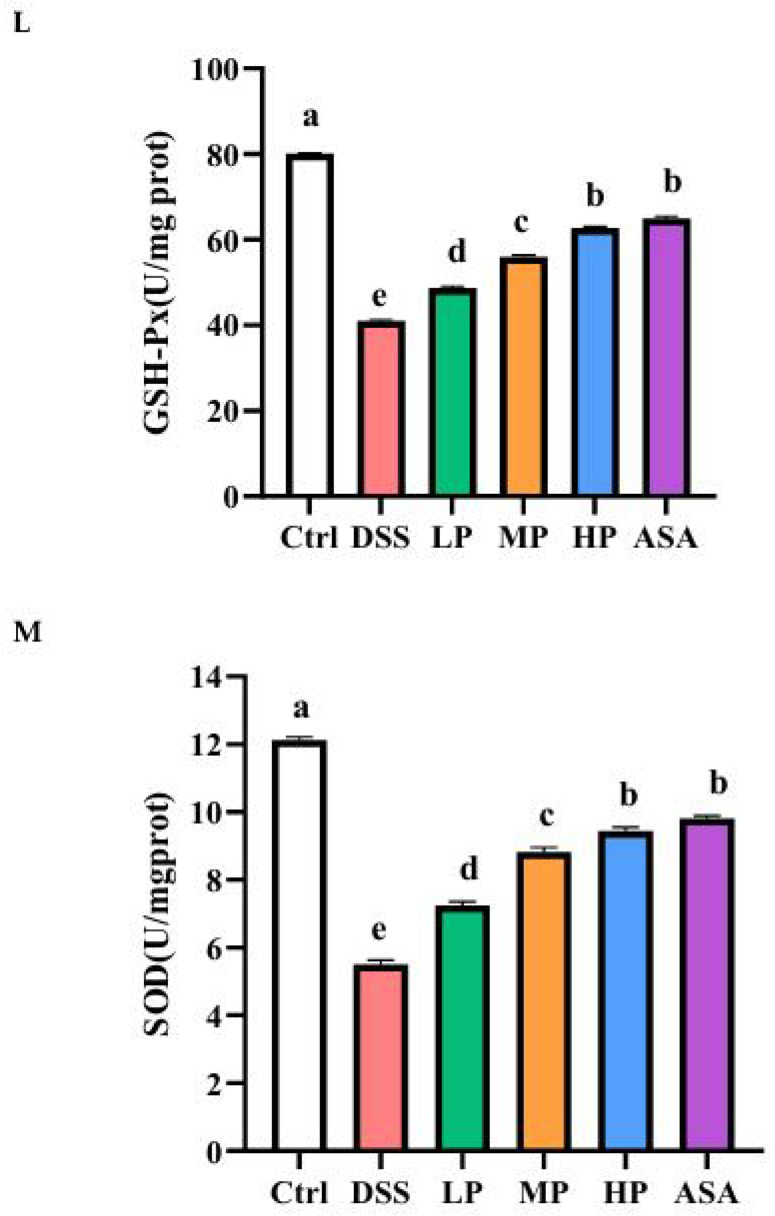
Relief effect of postbiotic Pa JY062 on 3% dextran sodium sulfate (DSS)-induced colonic barrier injury (*n* = 3). (**A**) Evans blue permeability. (**B**) Physical picture of Evans blue permeability. (**C**) Colon microstructure. (**D**) The mRNA expression of intestinal barrier proteins (Claudin-1, Occludin, ZO-1 and E-Cadherin). (**E**) Immunohistochemical images of Claudin-1. (**F**) Image J was used to quantify the immunohistochemical images of Claudin-1. (**G**) Immunohistochemical images of Occludin. (**H**) Image J was used to quantify the immunohistochemical images of Occludin. (**I**) Immunohistochemical images of ZO-1. (**J**) Image J was used to quantify the immunohistochemical images of ZO-1. (**K**) Representative AB-PAS pictures. (**L**) Glutathione peroxidase (GSH-Px). (**M**) Superoxide dismutase (SOD). The different letters indicate statistically significant differences (*p* < 0.05).

The integrity of the intestinal barrier is regulated by multiple factors. Therefore, we analyzed the protective effect of Pa JY062 on 3% DSS-induced colonic barrier damage from multiple perspectives, including Evans blue permeability, microstructure, expression of barrier regulatory proteins, and oxidative stress. Compared with the Ctrl group, the Evans blue permeability of the DSS group increased significantly (DSS: 0.1073 ± 0.005 vs. Ctrl: 0.048 ± 0.003, *p* < 0.01, Figure 5A,B). TEM revealed that the structural integrity of the intestinal barrier junctions was compromised, accompanied by a loss of the brush border (Figure 5C). RT-qPCR exhibited the expression of barrier junction proteins (ZO-1, Occludin, Claudin-1 and E-cadherin) decreased (Figure 5D). Immunohistochemistry (IHC) analyses further demonstrated that the Ctrl group exhibited the strongest positive staining (brown-yellow) for ZO-1, Occludin, and Claudin-1, whereas the DSS group showed a substantial reduction in the expression of these proteins (Figure 5E–J). Meanwhile, the number of goblet cells decreased (Figure 5K), and the enzyme activities of GSH-Px (DSS: 40.94 ± 0.34 U/mg prot vs. Ctrl: 80.12 ± 0.17 U/mg prot, *p* < 0.01, Figure 5L) and SOD (DSS: 5.5 ± 0.13 U/mg prot vs. Ctrl: 12.11 ± 0.10 U/mg prot, *p* < 0.01, Figure 5M) decreased significantly. The protective effect of Pa JY062 against DSS-induced intestinal barrier damage was dose-dependent, with the most pronounced amelioration observed in the high-dose (HP) group. The Evans blue permeability in the HP group was markedly reduced compared to the DSS group (HP: 0.0547± 0.0021 vs. DSS: 0.1073 ± 0.005, *p* < 0.01, Figure 5A,B), the colonic microvilli showed neat brush-like edges, which was similar to the ASA and Ctrl groups (Figure 5C), the expression of barrier junction proteins increased (Figure 5D, *p* < 0.01), the number of goblet cells increased (Figure 5K), and the enzyme activities of GSH-Px (HP: 62.74 ± 0.32 U/mg prot vs. DSS: 40.94 ± 0.34 U/mg prot, *p* < 0.01, Figure 5L) and SOD (HP: 9.43 ± 0.11 U/mg prot vs. DSS: 5.5 ± 0.13 U/mg prot, *p* < 0.01, Figure 5M) increased significantly. In conclusion, Pa JY062 alleviated DSS-induced intestinal damage by restoring microvilli architecture, enhancing the expression of junctional proteins, and reducing permeability, suggesting its therapeutic potential for maintaining intestinal homeostasis.

### 3.6. Pa JY062 Alleviated the Inflammatory Response Induced by DSS in Mice

**Figure 6:**
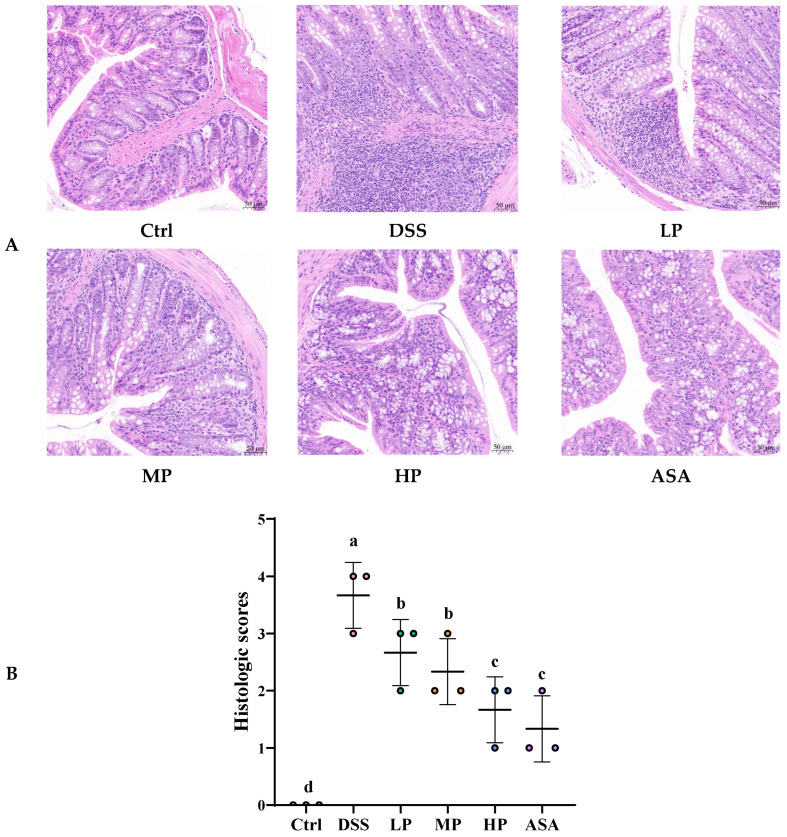

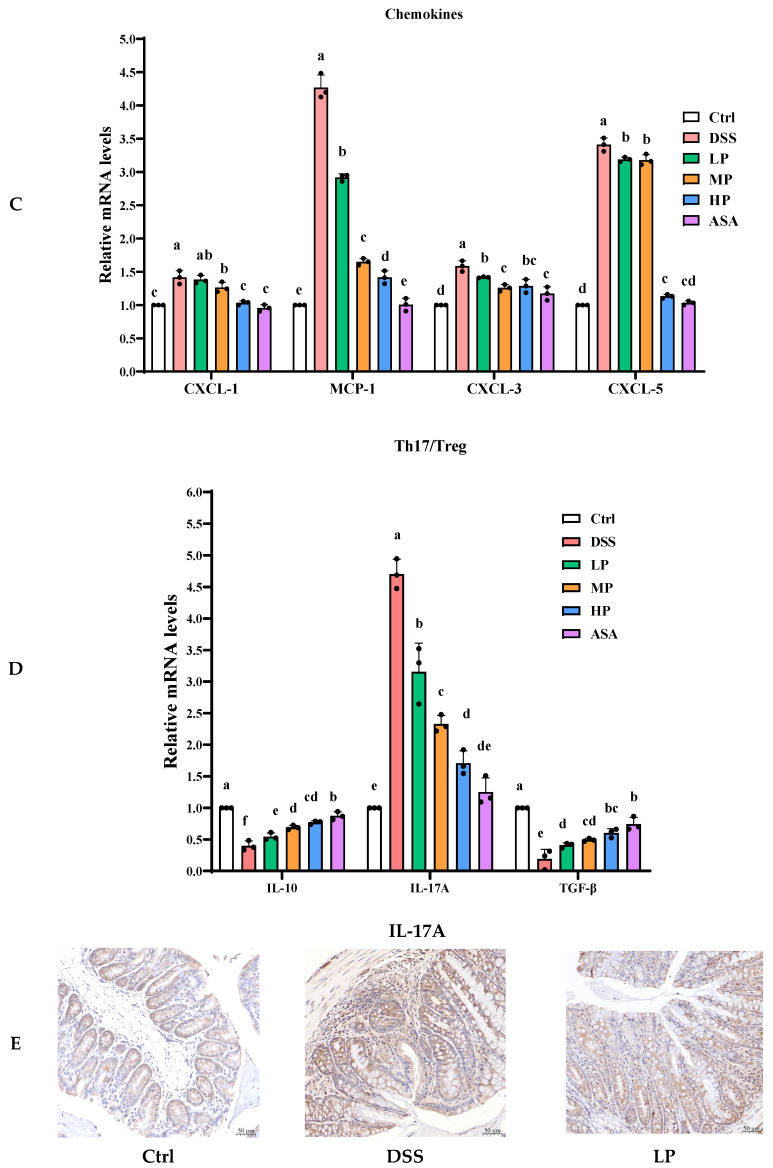

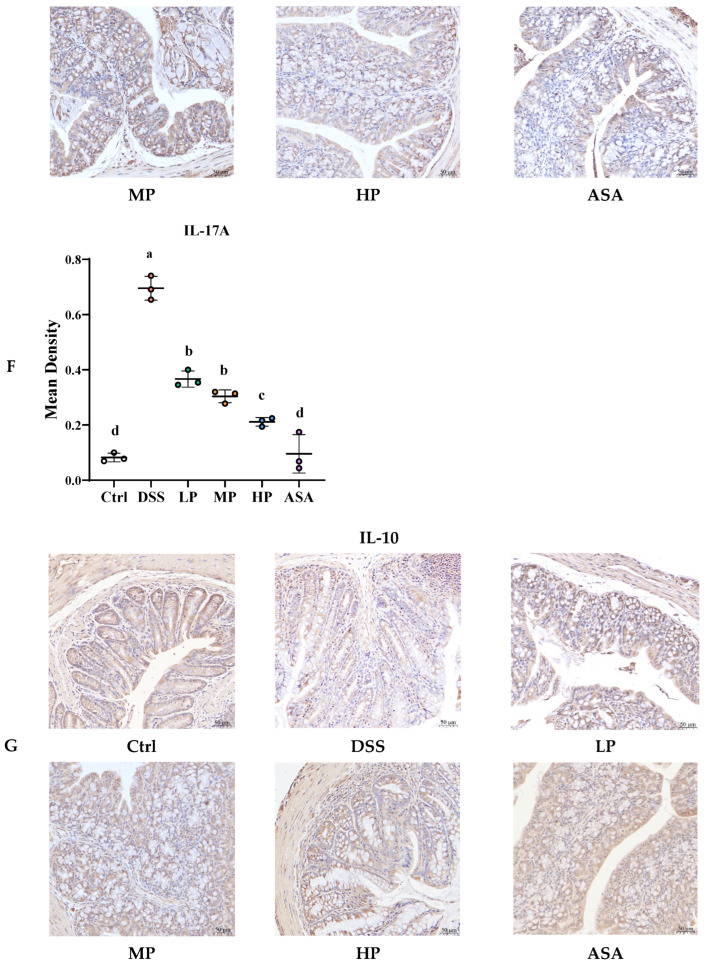

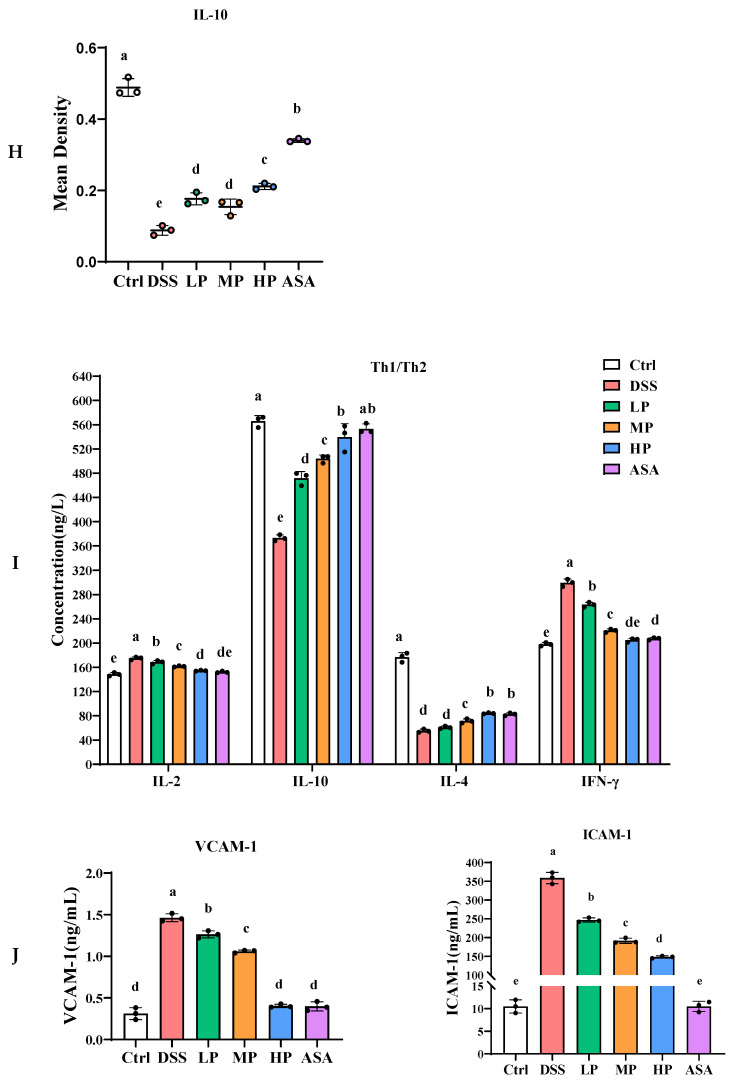
Pa JY062 alleviated the gut microbiota induced by 3% DSS in mice (*n* = 3). (**A**) H&E staining. (**B**) Histologic scores. (**C**) Relative mRNA levels of chemokines. (**D**) The changes in Th17/Treg balance. (**E**) Immunohistochemical images of IL-17A. (**F**) Image J was used to quantify the immunohistochemical images of IL-17A. (**G**) Immunohistochemical images of IL-10. (**H**) Image J quantification of protein expression of IL-10. (**I**) The changes in Th1/Th2 balance. (**J**) Adhesion molecule analysis. The different letters indicate statistically significant differences (*p* < 0.05).

UC involves a self-amplifying inflammatory cascade characterized by inflammatory infiltration, release of pro-inflammatory factors, and dysregulation of T-helper (Th) cell responses. Here, we investigated the multi-faceted mechanisms by which Pa JY062 interrupts this vicious cycle. H&E staining revealed severe mucosal damage in DSS-treated mice, characterized by crypt loss, glandular architecture disruption, and inflammatory infiltration into the basal layer (Figure 6A). Pa JY062 dose-dependently restored crypt integrity, increased goblet cell density, and attenuated leukocyte infiltration, with the HP group showing maximal efficacy. Consistent with these observations, the histopathological score of colon tissue was significantly lower in the HP group compared to the DSS group (Figure 6B, *p* < 0.01).

The transcript levels of chemokines demonstrated DSS-induced overexpression of *CXCL-1* (1.42 ± 0.1 fold), MCP-1 (4.27 ± 0.19 fold), *CXCL-3* (1.59 ± 0.08 fold), and *CXCL-5* (3.41 ± 0.1 fold) versus Ctrl. Both ASA and Pa JY062 decreased chemokine hyperactivation (HP group reductions: *CXCL-1* 27%, *MCP-1* 67%, *CXCL-3* 19%, *CXCL-5* 67%; *p* < 0.01; Figure 6C).

The DSS group exhibited Th17/Treg imbalance, marked by elevated *IL-17A* (4.7-fold vs. Ctrl) and suppressed *IL-10* (0.4-fold) and *TGF-β* (0.2-fold). Pa JY062 restored cytokine equilibrium (HP group: *IL-17A* 0.4-fold, IL-10 1.9-fold, *TGF-β* 3.2-fold vs. DSS; *p* < 0.001; Figure 6D). IHC confirmed these findings, and the DSS group showed intense IL-17A immunostaining, whereas HP treatment reduced IL-17A positivity and enhanced IL-10 expression (Figure 6E–H).

ELISA further revealed a Th1/Th2 imbalance in DSS mice, indicated by significantly elevated serum levels of the Th1 cytokines IL-2 (174.89 ± 2.43 ng/L) and IFN-γ (299.44 ± 6.10 ng/L) compared to the Ctrl group (148.50 ± 2.82 and 197.89 ± 2.92 ng/L, respectively; *p* < 0.01). Pa JY062 normalized Th1 cytokines (HP group: IL-2 58 ± 6 ng/L, IFN-γ 35 ± 4 ng/L; *p* < 0.05) and elevated Th2 mediators (IL-4: 84.09 ± 1.01 ng/L; IL-10: 539.63 ± 22.03 ng/L vs. DSS 55.20 ± 2.72 and 373.08 ± 5.02 ng/L; *p* < 0.001; Figure 6I).

Adhesion molecule analysis showed DSS-induced upregulation of ICAM-1 (34.17-fold) and VCAM-1 (4.68-fold) compared with Ctrl, which Pa JY062 dose-dependently suppressed (HP group: ICAM-1 0.41-fold, VCAM-1 0.28-fold vs. DSS; *p* < 0.001; Figure 6J).

Collectively, these results demonstrate that Pa JY062 alleviates DSS-induced colitis by suppressing leukocyte infiltration, rebalancing cytokine homeostasis, and rectifying T-helper cell dysregulation.

### 3.7. Pa JY062 Alleviated DSS-Induced Dysbiosis of the Intestinal Microbiota and Depletion of SCFAs in Mice

**Figure 7:**
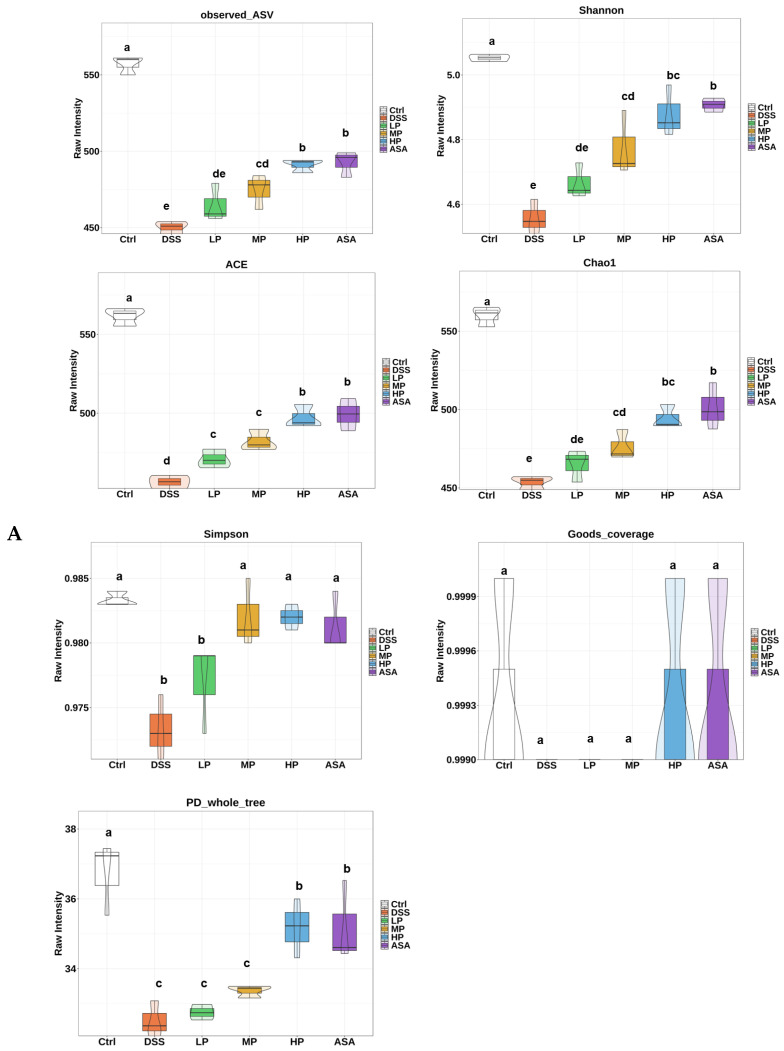

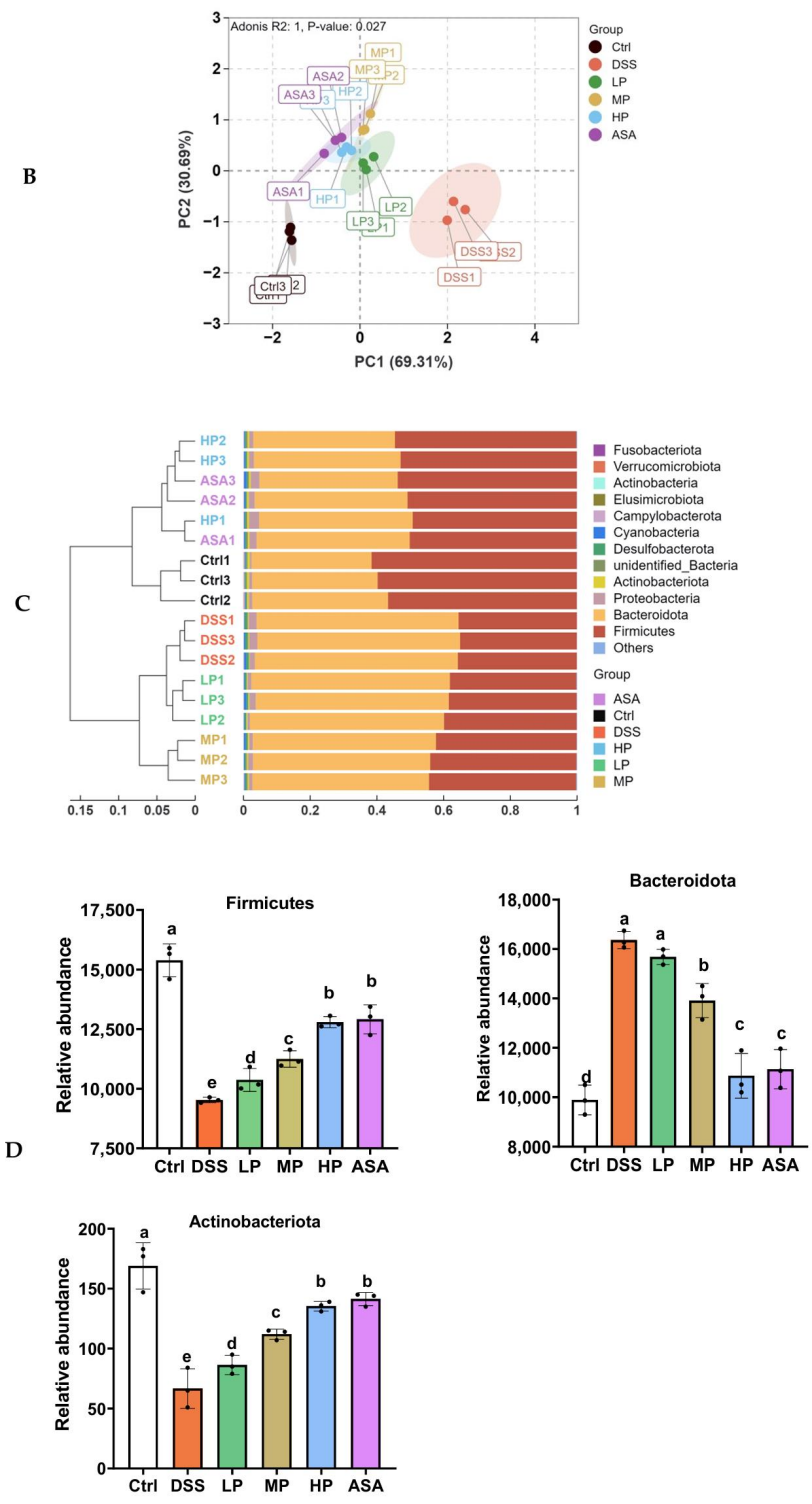

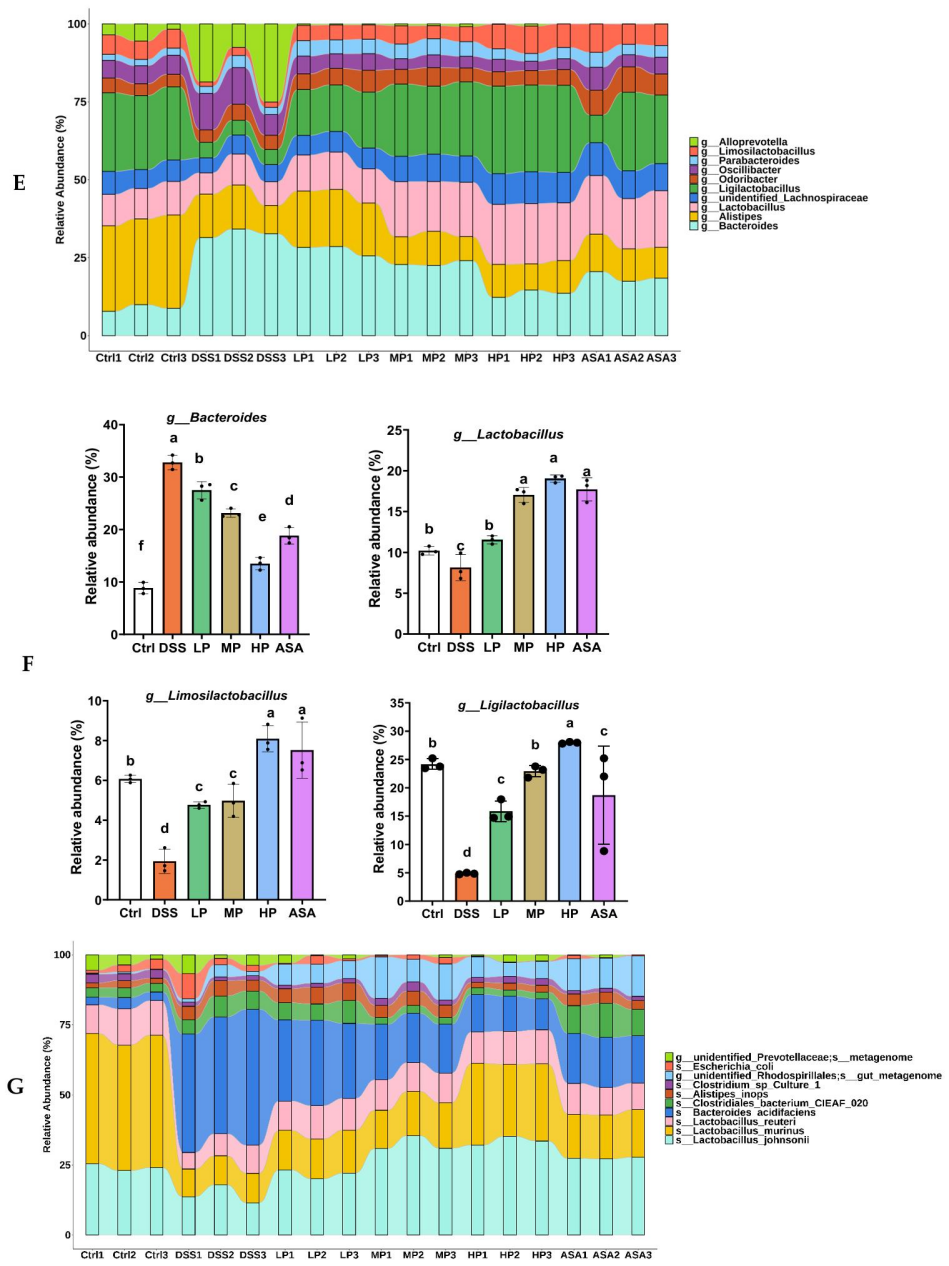

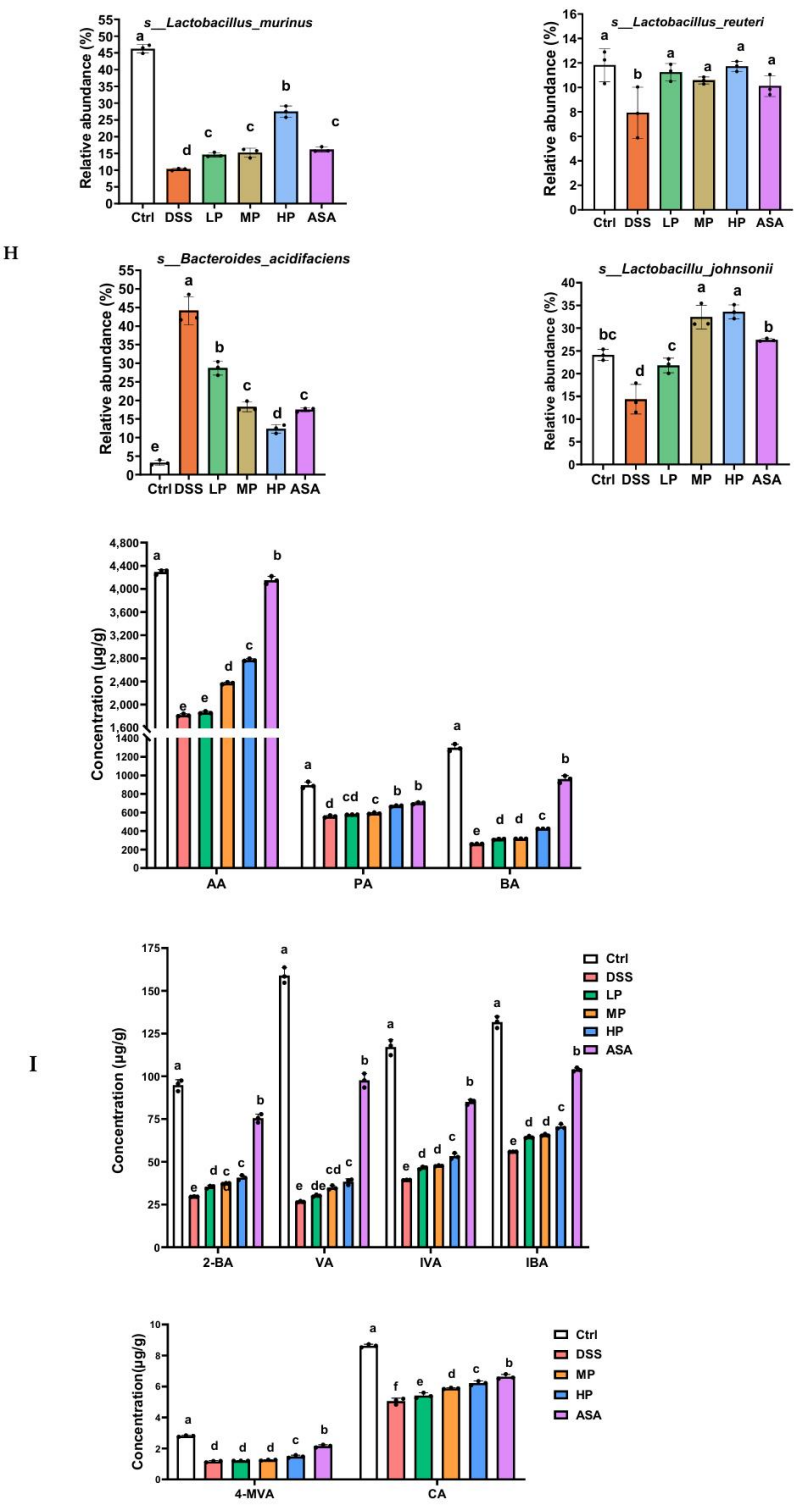
Pa JY062 alleviated the imbalance of intestinal microbiota homeostasis induced by 3% DSS in mice (*n* = 3). (**A**) Alpha diversity analysis indices (Observed_ASV, Shannon, Simpson, Chao1, ACE, Goods_coverage, PD_whole_tree). (**B**) Principal component analysis (PCA) of beta diversity. (**C**) Unweighted pair-group method with arithmetic mean (UPGMA) clustering tree. (**D**) Relative abundance of Firmicutes, Bacteroidetes, and Actinobacteriota based on the UPGMA clustering tree. (**E**) Relative abundance at genus level. (**F**) Relative abundance of significantly different genera. (**G**) Relative abundance at species level. (**H**) Relative abundance of significantly different species. (**I**) Short-chain fatty acid analysis.The different letters indicate statistically significant differences (*p* < 0.05).

To comprehensively assess the modulatory influence of Pa JY062 on UC in mice, we investigated the changes in gut microbiota and SCFAs. The alpha diversity analysis (Figure 7A) indicated that the DSS group displayed the lowest indices of species richness (Observed ASV, Shannon, Simpson, Chao1, ACE) compared to the Ctrl group. Pa JY062 treatment dose-dependently restored these indices, with the HP group showing the most pronounced restoration (*p* < 0.05). The phylogenetic diversity index (PD whole tree) followed a similar trend, while goods coverage remained consistent across groups, confirming adequate sequencing depth. Beta diversity analysis via principal component analysis (PCA) demonstrated distinct clustering between the DSS and Ctrl groups. In contrast, the LP, MP, HP, and ASA groups exhibited closer proximity to the Ctrl group, with intra-group homogeneity, indicating Pa JY062 effectively mitigated DSS-induced beta diversity disruption (Figure 7B).

At the phylum level, DSS treatment significantly altered the microbial composition, notably increasing the relative abundance of Bacteroidota and decreasing that of Firmicutes and Actinobacteriota compared to the Ctrl group (*p* < 0.01; Figure 7C,D). These alterations were reversed in the HP group, achieving a profile comparable to the ASA group (Figure 7C,D).

At the genus level, Pa JY062 (HP) significantly ameliorated the DSS-induced depletion of beneficial genera including *Limosilactobacillus* (4.18-fold), Lactobacillus (2.34-fold), and *Ligilactobacillus* (5.74-fold) (*p* < 0.05). Concurrently, it reduced the overgrowth of Bacteroides (DSS:32.78% vs. HP: 13.49%, *p* < 0.01) (Figure 7E,F). Species-level analysis further confirmed that HP treatment enhanced the abundance of *Lactobacillus murinus* (2.68-fold), *Lactobacillus johnsonii* (2.34-fold), and *Limosilactobacillus reuteri* (1.47-fold), while suppressing *Bacteroides acidifaciens* (27% reduction vs. DSS, *p* < 0.01; Figure 7G,H).

SCFAs quantification revealed that DSS treatment drastically decreased fecal acetate (AA: 1819.99 ± 22.83 vs. Ctrl 4292.28 ± 41.49 μg/g), propionate (PA: 556.65 ± 10.68 vs. 893.81 ± 34.60 μg/g), and butyrate (BA: 260.11 ± 2.42 vs. 1299.15 ± 38.11 μg/g) (*p* < 0.001). HP treatment restored these levels (AA: 2772.70 ± 21.08, PA: 669.84 ± 6.79, BA: 324.14 ± 0.42 μg/g) and significantly increased 2-methylbutyrate (2-BA), valerate (VA), and caproate (CA) (*p* < 0.01 vs. DSS) (Figure 7I).

### 3.8. Correlation Analysis Between Gut Microbiota and Intestinal Homeostasis

**Figure 8:**
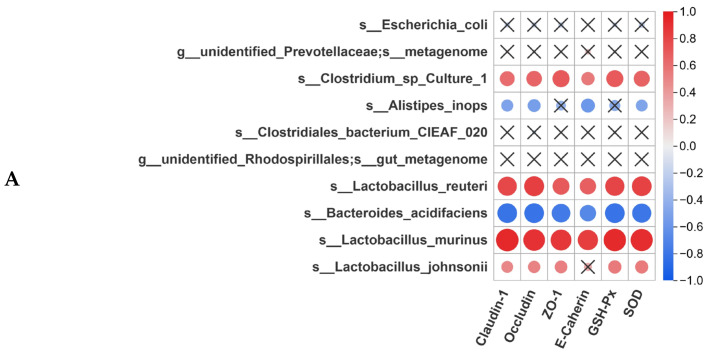

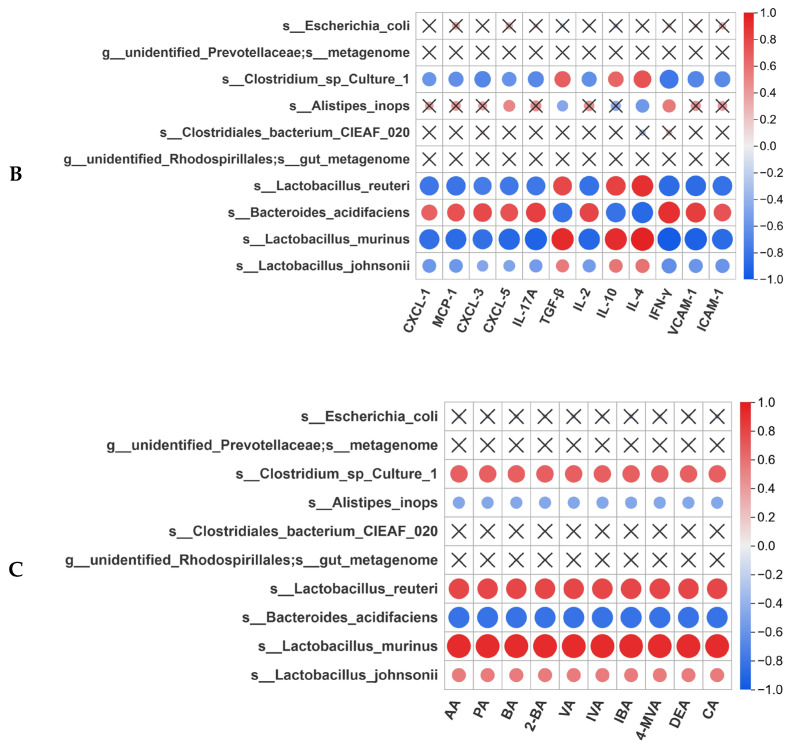
Spearman correlation analysis between species-level abundance and short-chain fatty acids, intestinal barrier proteins, and inflammatory factors.(**A**) Correlation between key gut barrier markers and gut microbiota. (**B**) Correlation between intestinal inflammation markers and gut microbiota. (**C**) Correlation between short-chain fatty acids (SCFA) and gut microbiota. In the panels, the ‘X’ symbol indicates no significant association; red and blue circles denote positive and negative correlations, respectively; the size of the circle represents the strength of the correlation.

To elucidate the interplay among gut microbiota, intestinal barrier function, inflammatory factors, and SCFAs, Spearman correlation analysis was performed on 10 key microbial species, intestinal barrier proteins (Claudin-1, Occludin, ZO-1, E-Cadherin), antioxidant enzymes (GSH-Px, SOD), inflammatory factors, and SCFA levels. Claudin-1, Occludin, ZO-1, E-Cadherin, GSH-Px, and SOD exhibited strong positive correlations with *L. murinus* (r = 0.93–0.97, *p* < 0.01) and *L. reuteri* (r = 0.73–0.88, *p* < 0.01). These markers showed significant negative correlations with *B. acidifaciens* (r = −0.87 to −0.69, *p* < 0.01) (Figure 8A).

Pro-inflammatory factors (CXCL-1, MCP-1, CXCL-3, CXCL-5, IL-2, IL-17A, IFN-γ, VCAM-1 and ICAM-1) were negatively associated with *L. murinus* (r = −0.93 to −0.81) and *L. reuteri* (r = −0.77 to −0.69), while positively linked to *B. acidifaciens* (r = 0.63–0.88) (*p* < 0.001). Anti-inflammatory cytokines (IL-4, IL-10, TGF-β) were positively correlated with *L. murinus* (r = 0.92–0.90) and *L. reuteri* (r = 0.63–0.88), while negatively correlated with *B. acidifaciens* (r = −0.89 to −0.81) (*p* < 0.01) (Figure 8B).

SCFAs concentrations positively correlated with *L. murinus* (r = 0.91–0.95) and *L. reuteri* (r = 0.81–0.85), but negatively with *B. acidifaciens* (r = −0.94 to −0. 89) (*p* < 0.01 for all) (Figure 8C). These results indicate that DSS-induced colitis disrupts the proliferation of beneficial lactobacilli (*L. murinus*, *L. reuteri*) and promotes the expansion of *B. acidifaciens*. These collective changes are associated with impaired intestinal barrier integrity and antioxidant capacity. High-dose Pa JY062 likely alleviates colitis by restoring probiotic (*L. murinus*, *L. reuteri*), enhancing SCFAs production, and rebalancing pro-/anti-inflammatory cytokine networks (Th1/Th2 and Th17/Treg balance).

## 4. Discussion

UC is a multifactorial intestinal disorder mechanistically linked to excessive oxidative stress [16], compromised epithelial barrier integrity, dysregulated inflammatory cascades, and gut microbiota dysbiosis [17]. Our findings demonstrated postbiotic Pa JY062 alleviated colitis through multidimensional restoration. Pa JY062 exhibits potent antioxidant efficacy across in vitro and in vivo systems. It displayed exceptional radical scavenging capacity in vitro, as evidenced by its DPPH radical scavenging activity and total antioxidant capacity (T-AOC) (Figure 1A,B), while also maintaining low intracellular ROS levels in Caco-2 cells (Figure 2A,B). In vivo, Pa JY062 administration significantly enhanced colonic antioxidant defenses in DSS-treated mice, elevating the activities of SOD and GSH-Px (Figure 5L,M).

The DPPH radical scavenging rate of the Pa JY062 postbiotic (36.54 ± 2.9%) was significantly higher than that of other reported postbiotics, including a lysed cell preparation of *Levilactobacillus brevis* BK3 (30.97 ± 5.56%) [18] and a cell-free fermented liquid of *Weissella cibaria* ETE (35.65 ± 2.08%) [19]. This enhanced antioxidant activity can be attributed to the unique composite nature of Pa JY062. Unlike the singular compositions of the comparator postbiotics, Pa JY062 is a powdered product comprising the entire fermented material containing both inactivated bacterial cells and metabolites from the skim milk medium. We propose that this integrative composition results in a synergistic interplay among its dominant components (Table A5, Figure 3), leading to a more potent and multifaceted antioxidant capacity. Emerging evidence indicates that various organic acid metabolites, including allantoin [20], citric acid [21], 3-phenyllactic acid [22], salicylic acid [23], quinic acid [24], and stachydrine hydrochloride [25], have antioxidant properties. Furthermore, as vital constituents of Pa JY062, histidine [26], L-norvaline [27], phenylalanine [28], glycine [29], tryptophan [30], threonine [31], and serine [32] have been observed in prior studies to possess significant antioxidant activity. Furthermore, the efficacy of Pa JY062 may be augmented by its intrinsic betaine content. Betaine is a well-established osmoprotectant that safeguards intestinal epithelial cells by maintaining ion homeostasis [33]. Importantly, it also exhibits anti-inflammatory properties by inhibiting the NF-κB pathway, subsequently suppressing the expression of pro-inflammatory mediators including IL-1β, COX-2, and iNOS [34].

Since oxidative stress can damage the intestinal mucosa, we further investigated the protective effects of Pa JY062. Our findings demonstrate that 800 mg/kg Pa JY062 integrates epithelial restitution, ultrastructural repair, and junctional reinforcement into a coordinated action (Figure 5), which collectively underlies its therapeutic effect against UC. For a mouse dose of 800 mg/kg, the HED is approximately 65 mg/kg, equating to a total daily dose of 3.9 g for a 60 kg adult. A previous animal study utilizing postbiotics from *Lactobacillus helveticus* KLDS 1.8701 reported an effective dose of 1200 mg/kg/d in mice, which translates to a human-relevant dose of approximately 6 g/day [35]. A randomized, double-blind, crossover clinical trial demonstrated the safety and efficacy of a postbiotic preparation (Probio-Eco^®^) administered at a dose of 7.2 g/day for 21 days [36]. This clinical dose is significantly higher than our calculated HED of ~4 g/day, providing strong evidence for the practical acceptability and safety of our proposed dosage range in humans.

The preservation of intestinal barrier integrity is intricately associated with the accurate migration of epithelial cells [37]. Our results demonstrate that Pa JY062 not only ameliorated the DSS-induced damage and loss of microvilli (Figure 5C), but also enhanced intestinal barrier integrity by upregulating the expression of tight junction proteins (ZO-1, Occludin, Claudin-1) and the adherens junction protein E-cadherin (Figure 5D–J), while reducing Evans blue permeability (Figure 5A,B). Microvilli restoration is not merely structural, but functionally pivotal. Increasing the density and length of microvilli expands the surface area of the apical membrane, enhancing the nutrient absorption of mature intestinal epithelial cells [38]. The physical barrier, constituting the architectural core of intestinal barrier integrity, comprises polarized enterocytes interconnected via specialized junctional complexes—including TJs, AJs, and desmosomes [39]. While intestinal epithelial cells govern transcellular permeability through selective solute/water transport, TJs localized at the apical intercellular membrane domains serve as gatekeepers of paracellular permeability by forming size- and charge-selective ion channels [40]. AJs, primarily mediated by E-cadherin, orchestrate intestinal epithelial cell adhesion dynamics, epithelial restitution through coordinated proliferation/migration and barrier maintenance [41]. Notably, *Lactococcus lactis* HF08-derived postbiotic (P-HF08) [42], *Lacticaseibacillus paracasei* SNB-derived postbiotic [43], and *L. reuteri* DS0384 [44] also have the effects of promoting intestinal epithelial cell proliferation and repairing intestinal barrier damage. These parallels suggest a conserved barrier-repair capacity among phylogenetically distinct strains.

The compromised intestinal barrier in UC initiates a self-reinforcing inflammatory loop. Luminal pathogen translocation activates lamina propria macrophages, which secrete CXCL-1 and CXCL-5 (Figure 6C) to recruit neutrophils via ICAM-1/VCAM-1-mediated adhesion (Figure 6J). This cascade is further amplified by DSS-induced epithelial damage (Figure 6A,B). While neutrophils rapidly clear pathogens, their prolonged activation exacerbates mucosal injury through ROS overproduction [45], creating a niche for monocyte-derived macrophages to perpetuate inflammation via IL-1β. Notably, DSS disrupts the physiological inflammation–resolution axis, skewing immunity toward Th17-dominant acute inflammation (IL-17A up 4.7-fold, Figure 6D) that transitions to Th1-driven chronicity (IFN-γ increased, Figure 6I). IFN-γ binding to interferon-γ receptor (IFNGR) triggers a Janus kinase (JAK)-signal transducer and activator of transcription 1 (STAT1) signaling cascade, where JAK phosphorylates STAT1, thereby guiding macrophage polarization toward the pro-inflammatory M1 phenotype [46]. These alterations were clinically manifested as colonic shortening (Figure 4H,I), splenomegaly, and thymic involution (Figure 4F,G), reflecting systemic immune exhaustion and immunosuppression [47]. Our study elucidates that the 67% reduction in MCP-1 and CXCL-5 (Figure 6C) aligns with Pa JY062’s high salicylic acid, which inhibits NF-κB translocation by blocking IκBα phosphorylation [48]. 5-ASA is a salicylic acid derivative with anti-inflammatory properties [49]. Notably, Pa JY062 had similar effects to 5-ASA in suppressing CXCL-1/CXCL-3 (Figure 6C). Pa JY062 selectively suppressed Th17 responses (decreased IL-17A) while promoting Treg maintenance, as evidenced by elevated TGF-β levels (Figure 6D). It also fostered the differentiation of monocytes into regulatory macrophages (increased IL-10), thereby rectifying the Th17/Treg imbalance and resolving acute inflammation (Figure 6E). These findings are consistent with our previous report [10]. The clinical potential of Pa JY062 is supported by favorable comparisons with natural products that have established efficacy in UC management. For instance, in our model, Pa JY062 induced IL-10 levels (~540 ng/L) substantially exceeding those reported for curcumin (~120 ng/L) [50], which has demonstrated clinical benefits in mild-to-moderate UC [51]. Furthermore, the efficacy of natural indigo (IN) in clinical trials [52], and its mechanistic role in suppressing macrophage inflammation and MCP-1 production in mice [53], align with our finding that Pa JY062 significantly reduces colonic MCP-1 (Figure 6C). Given that Pa JY062 outperforms a clinically relevant benchmark (curcumin) in a key immunoregulatory aspect and shares a mechanistic pathway with another effective agent (IN), we posit that Pa JY062 holds significant promise for clinical translation in UC therapy.

The Pa JY062 group exhibited concurrent reduction in Th1-associated cytokines (IFN-γ and IL-2; Figure 6I) alongside an increase in Th2 markers (IL-4 and IL-10), indicating a potential shift from cell-mediated toward humoral immunity during intestinal inflammation. This rebalancing of the Th1/Th2 axis likely represents a compensatory anti-inflammatory response to mucosal damage, wherein the well-established immunosuppressive cytokine IL-10 functions to limit excessive inflammation [54]. The parallel suppression of ICAM-1/VCAM-1 (Figure 6J) further highlights its endothelial-protective role. The suppression of ICAM-1 and VCAM-1 by Pa JY062 (Figure 6J) likely confers similar therapeutic benefits: reduced VCAM-1 attenuates α4 integrin-mediated immune cell recruitment [55], while ICAM-1 inhibition suppresses neutrophil infiltration and promotes anti-inflammatory macrophage polarization [56], collectively mitigating chronic inflammation.

Meanwhile, our study provides evidence that Pa JY062 dose-dependently restores DSS-induced gut microbiota dysbiosis. The high-dose intervention not only normalized alpha and beta diversity indices (Figure 7A,B), but also reversed specific pathogenic shifts at the phylum level, namely the increased Bacteroidota/Firmicutes ratio (Figure 7C,D), a signature commonly observed in clinical UC patients [57,58]. A particularly noteworthy finding was the 5.74-fold increase in *Ligilactobacillus* (Figure 7E,F), a genus with well-documented anti-colitic properties. Notably, *Ligilactobacillus salivarius* CCFM 1266 [59], *Ligilactobacillus salivarius* Li01 [60], and *Ligilactobacillus acidipiscis* YJ5 [61] demonstrated significant therapeutic efficacy in alleviating colitis. Concurrently, *Limosilactobacillus* and *Lactobacillus* exhibited significant increases. Species-level analysis further identified *L. murinus* (2.68-fold), *L. johnsonii* (2.34-fold), and *L. reuteri* (1.47-fold) as the predominant species enhanced by Pa JY062 treatment (Figure 7G, H). The dominance of *Lactobacillus johnsonii* in HP group correlates with its unique capacity to polarize resident macrophages toward an immunoregulatory CD206+ phenotype and mediates IL-10 secretion via the TLR1/2-STAT3 signaling axis to ameliorate experimental colitis [62]. Crucially, thermal inactivation of *L. johnsonii* abolished its therapeutic efficacy in ameliorating murine colitis [63]. *L. reuteri* potentiated PD-1+ T follicular helper (Tfh) cell-dependent IgA responses, thereby restructuring the gut microbiota (e.g., *Akkermansia muciniphila*, AKK) and ameliorating DSS- triggered colitis and microbial imbalance [64,65]. Consistent with our findings, prior studies have demonstrated that combinatorial administration of LGG with an anti-PD-1 antibody synergistically enhanced the abundance of *L. murinus*. Notably, *L. murinus* was mechanistically linked to the activation of dendritic cells (DCs) in the murine intestinal microenvironment [66], indicating that *L. murinus* is important for the restoration of intestinal immunity in colitis. Hence, *L. murinus*, *L. johnsonii*, and *L. reuteri* play critical roles in Pa JY062-regulated colitis. SCFAs, which are metabolites produced by the gut microbiota, vary in concentration in response to alterations in microbial composition. We observed a recovery of SCFAs, particularly butyrate (from 260.11 to 324.14 μg/g; Figure 7I). The restoration of butyrate, which synergistically contributes to intestinal homeostasis [67], likely links the Pa JY062-induced microbial shifts to the observed barrier repair.

Our systematic correlation analysis establishes that Pa JY062 achieves multimodal colitis amelioration through precision remodeling of the “gut microbiota–metabolite–oxidative stress-barrier–immunity network”, distinguishing it from single-target anti-inflammatory or antibacterial strategies. *L. murinus* serves as a pivotal bacterium for mucosal restoration. Its abundance exhibits a robust positive correlation with barrier proteins (Claudin-1/ZO-1, r = 0.93–0.97) and antioxidant markers (Figure 8A), implying that this strain may mitigate mucosal injury by reinforcing mucus layer integrity and scavenging intestinal free radicals. This aligns with documented Lactobacillus mechanisms, including antioxidants and the modulation of the Nrf2 antioxidant pathway [68,69]. The strong correlation of both *L. reuteri* and *L. murinus* with the upregulation of tight junction proteins (ZO-1, Occludin) and E-cadherin (Figure 8A) suggests they may act in concert to reinforce the epithelial barrier.

The strong negative correlation between *L. murinus* abundance and pro-inflammatory factors (e.g., IL-17A and IFN-γ; r = −0.93 to −0.81, Figure 8B) suggests its potential as a quantifiable biomarker for monitoring inflammatory activity. This finding provides a new perspective for clinical prediction of inflammatory status by detecting the abundance of commensal bacteria, which is could offer a more gut-specific assessment than measuring systemic inflammatory markers in serum. The strong positive correlation between SCFAs and *L. murinus*/*L. reuteri*, coupled with the negative correlation with *B. acidifaciens* (Figure 8C), suggests a positive ecological feedback loop: Pa JY062 promotes beneficial *lactobacilli*, which produce SCFAs that in turn inhibit pathogens and further foster a healthy microbial environment. This self-reinforcing cycle may represent an advantage over some traditional probiotics, potentially moving beyond a simple “occupancy effect” by actively reshaping the microbial niche through metabolic output. Pa JY062’s therapeutic efficacy arises not from isolated mechanisms but through a trans-hierarchical cascade: *Lactobacillus* enrichment, oxidative stress mitigation, barrier restoration, SCFA elevation, anti-inflammatory resolution. This systems-level mode of action establishes a novel paradigm for IBD management. Critically, such multi-dimensional postbiotic targeting may mitigate therapeutic escape and systemic toxicity risks inherent to single-pathway pharmacotherapies.

While our findings provide strong preclinical evidence for the efficacy of postbiotic Pa JY062, several limitations inherent to our experimental models must be acknowledged in terms of clinical translation. First, the DSS-induced colitis model, while excellent for studying acute epithelial injury and inflammation, does not fully recapitulate the chronic, relapsing–remitting nature and complex immune dysregulation of human UC. Second, the Caco-2 monoculture system, although valuable for initial barrier assessment, lacks the immune compartment and microbiome cues of the human gut, limiting the full validation of the immunomodulatory mechanisms proposed. These limitations are compounded by the inherent complexity of the postbiotic mixture itself, which precludes attributing effects to a single constituent—a common challenge in postbiotic research.

To bridge this translational gap, we propose a clear pathway for future research, progressing from human-relevant systems like patient-derived organoids to well-controlled clinical trials in human subjects to assess safety, dosage, and therapeutic potential. Notwithstanding these limitations, our work establishes a robust foundational framework that justifies and guides these essential next steps toward clinical application.

## 5. Conclusions

This research illustrated that postbiotic Pa JY062 conferred multidimensional repair against DSS-induced colitis by regulating the balance of intestinal microecology. Specifically, the mRNA expression of intestinal barrier proteins (ZO-1, Claudin-1, Occludin and E-cadherin) were elevated, and the activities of antioxidant enzymes (GSH-Px, SOD) in the colon were increased, it exhibited a mitigating influence on the symptoms of colitis in mice. Meanwhile, it also decreases the infiltration of inflammatory, restoring cytokine homeostasis, and correcting Th cell dysregulation (Th1/Th2 and Treg/Th17). Further analyses indicated that Pa JY062 selectively promotes the proliferation of beneficial bacteria (*L. murinus* and *L. reuteri*) while suppressing *B. acidifaciens* growth, concomitant with increased SCFAs production. These findings provide compelling evidence for the probiotic potential of postbiotics in gut health and pave the way for broader therapeutic applications. However, current research remains largely confined to laboratory-scale investigations, with limited studies on large-scale industrial production optimization and cost-effectiveness. Addressing these gaps is crucial to facilitate the commercialization and widespread adoption of postbiotic-based interventions.

## Figures and Tables

**Table 1:** T500-targeted metabolomics identified 198 previously unreported components in PaJY062.

	Compounds	Class	Chemical Formula	Content (ng/mL)
1	2-thiocytidine	Nucleotide and its metabolomics	C_9_H_13_N_3_O_4_S	39,470.82
2	Uridine	Nucleotide and its metabolomics	C_9_H_12_N_2_O_6_	11,549.76
3	UMP	Nucleotide and its metabolomics	C_9_H_13_N_2_O_9_P	4668.834
4	Cytidine	Nucleotide and its metabolomics	C_9_H_13_N_3_O_5_	3045.108
5	Uracil	Nucleotide and its metabolomics	C_4_H_4_N_2_O_2_	2854.398
6	Guanosine	Nucleotide and its metabolomics	C_10_H_13_N_5_O_5_	2787.54
7	Xanthosine	Nucleotide and its metabolomics	C_10_H_12_N_4_O_6_	2771.946
8	Inosine	Nucleotide and its metabolomics	C_10_H_12_N_4_O_5_	2457.132
9	5-carboxycytidine	Nucleotide and its metabolomics	C_10_H_13_N_3_O_7_	2209.53
10	5′-deoxy-5′-methylthioadensine	Nucleotide and its metabolomics	C_11_H_15_N_5_O_3_S	2207.274
11	N4-acetyl-2′-O-methylcytidine	Nucleotide and its metabolomics	C_12_H_17_N_3_O_6_	1948.782
12	N6-threonylcarbamoyladenosine	Nucleotide and its metabolomics	C_15_H_20_N_6_O_8_	1913.694
13	5′-cytidylic acid	Nucleotide and its metabolomics	C_9_H_14_N_3_O_8_P	1047.882
14	IMP	Nucleotide and its metabolomics	C_10_H_13_N_4_O_8_P	1018.416
15	Adenosine	Nucleotide and its metabolomics	C_10_H_13_N_5_O_4_	805.098
16	AMP	Nucleotide and its metabolomics	C_10_H_14_N_5_O_7_P	663.306
17	Pseudouridine	Nucleotide and its metabolomics	C_9_H_12_N_2_O_6_	598.8162
18	2′-O-methylguanosine	Nucleotide and its metabolomics	C_11_H_15_N_5_O_5_	561.0024
19	2′-deoxycytidine	Nucleotide and its metabolomics	C_9_H_13_N_3_O_4_	318.0354
20	dCMP	Nucleotide and its metabolomics	C_9_H_14_N_3_O_7_P	301.2276
21	Guanine	Nucleotide and its metabolomics	C_5_H_5_N_5_O	299.766
22	2′-O-methyluridine	Nucleotide and its metabolomics	C_10_H_14_N_2_O_6_	254.6922
23	N,N-dimethylguanosine	Nucleotide and its metabolomics	C_12_H_17_N_5_O_5_	150.2892
24	5-hydroxymethylcytidine	Nucleotide and its metabolomics	C_10_H_15_N_3_O_6_	131.3736
25	Cytosine	Nucleotide and its metabolomics	C_4_H_5_N_3_O	101.3124
26	1-methylhistamine	Nucleotide and its metabolomics	C_6_H_13_C_l2_N_3_	79.2288
27	N2,N2,7-trimethylguanosine	Nucleotide and its metabolomics	C_13_H_19_N_5_O_5_	75.4542
28	5-hydroxymethyluracil	Nucleotide and its metabolomics	C_5_H_6_N_2_O_3_	59.51946
29	S-adenosyl-L-methioninate	Nucleotide and its metabolomics	C_15_H_22_N_6_O_5_S	58.54818
30	dTMP	Nucleotide and its metabolomics	C_10_H_15_N_2_O_8_P	54.00894
31	N6-methyladenosine	Nucleotide and its metabolomics	C_11_H_15_N_5_O_4_	49.44744
32	1-methyladenosine	Nucleotide and its metabolomics	C_11_H_15_N_5_O_4_	47.67612
33	7-methylguanosine	Nucleotide and its metabolomics	C_11_H_15_N_5_O_5_	30.29154
34	1-methylguanosine	Nucleotide and its metabolomics	C_11_H_15_N_5_O_5_	18.14586
35	N6, N6-dimethyladenosine	Nucleotide and its metabolomics	C_12_H_17_N_5_O_4_	17.21268
36	1-methylinosine	Nucleotide and its metabolomics	C_11_H_14_N_4_O_5_	2.482842
37	Adenine	Nucleotide and its metabolomics	C_5_H_5_N_5_	1.93587
38	Histidine	Amino acids	C_6_H_9_N_3_O_2_	85,330.8
39	L-norvaline	Amino acids	C_5_H_11_NO_2_	85,231.8
40	Phenylalanine	Amino acids	C_9_H_11_NO_2_	81,640.2
41	Sarcosine	Amino acids	C_3_H_7_NO_2_	77,844
42	Tyrosine	Amino acids	C_9_H_11_NO_3_	77,773.2
43	L-proline	Amino acids	C_5_H_9_NO_2_	73,215
44	L-methionine	Amino acids	C_5_H_11_NO_2_S	73,150.8
45	Glycine	Amino acids	C_2_H_5_NO_2_	71,337.6
46	Tryptophan	Amino acids	C_11_H_12_N_2_O_2_	61,059
47	L-leucine	Amino acids	C_6_H_13_NO_2_	59,032.8
48	L-isoleucine	Amino acids	C_6_H_13_NO_2_	49,271.58
49	Arginine	Amino acids	C_6_H_14_N_4_O_2_	19,382.1
50	Lysine	Amino acids	C_6_H_14_N_2_O_2_	13,724.82
51	Threonine	Amino acids	C_4_H_9_NO_3_	11,059.86
52	L-aspartate	Amino acids	C_4_H_7_NO_4_	10,520.7
53	Serine	Amino acids	C_3_H_7_NO_3_	7868.04
54	L-homocystine	Amino acids	C_8_H_16_N_2_O_4_S_2_	2382.066
55	L-homocitrulline	Amino acids	C_7_H_15_N_3_O_3_	1522.74
56	Homoserine	Amino acids	C_4_H_9_NO_3_	1503.816
57	Ornithine	Amino acids	C_5_H_12_N_2_O_2_	1352.352
58	L-asparagine	Amino acids	C_4_H_8_N_2_O_3_	1192.662
59	L-citrulline	Amino acids	C_6_H_13_N_3_O_3_	1018.074
60	H-Lys(Me)-OH chloride hydrochloride salt	Amino acids	C_9_H_21_ClN_2_O_2_	761.61
61	Homo-L-arginine	Amino acids	C_7_H_16_N_4_O_2_	331.8738
62	Allantoin	Organic acid and its derivatives	C_4_H_6_N_4_O_3_	99,550.2
63	Creatinine	Organic acid and its derivatives	C_4_H_7_N_3_O	61,650.6
64	4-aminobutyric acid	Organic acid and its derivatives	C_4_H_9_NO_2_	59,846.52
65	Hydroxyphenyllactic acid	Organic acid and its derivatives	C_9_H_10_O_4_	33,299.94
66	Creatine	Organic acid and its derivatives	C_4_H_9_N_3_O_2_	24,808.56
67	3-hydroxyisovaleric acid	Organic acid and its derivatives	C_5_H_10_O_3_	16,237.26
68	N-formylkynurenine	Organic acid and its derivatives	C_11_H_12_N_2_O_4_	13,108.98
69	Citric acid	Organic acid and its derivatives	C_6_H_8_O_7_	12,422.58
70	3-phenyllactic acid	Organic acid and its derivatives	C_9_H_10_O_3_	11,733.18
71	3-hydroxybutyric acid	Organic acid and its derivatives	C_4_H_8_O_3_	6365.04
72	2-hydroxyisovaleric acid	Organic acid and its derivatives	C_5_H_10_O_3_	6087.54
73	Potassium phenyl sulfate	Organic acid and its derivatives	C_6_H_5_KO_4_S	3206.172
74	Sebacic acid	Organic acid and its derivatives	C_10_H_18_O_4_	2042.904
75	Salicylic acid	Organic acid and its derivatives	C_7_H_6_O_3_	1362.336
76	Urocanic acid	Organic acid and its derivatives	C_6_H_6_N_2_O_2_	1306.572
77	Alpha-aminoadipic acid	Organic acid and its derivatives	C_6_H_11_NO_4_	848.568
78	Quinic acid	Organic acid and its derivatives	C_7_H_12_O_6_	782.604
79	Guanidinoacetic acid	Organic acid and its derivatives	C_3_H_7_N_3_O_2_	487.7532
80	Stachydrine hydrochloride	Organic acid and its derivatives	C_7_H_14_NO_2_	287.0406
81	2,4-Hexadienoic acid	Organic acid and its derivatives	C_6_H_8_O_2_	157.9614
82	Sumiki’s acid	Organic acid and its derivatives	C_6_H_6_O_4_	58.19304
83	Acetoacetic acid sodium salt	Organic acid and its derivatives	C_4_H_5_NaO_3_	56.4012
84	N-acetylputrescine	Organic acid and its derivatives	C_6_H_14_N_2_O	7.49262
85	Cyclamic acid	Organic acid and its derivatives	C_6_H_13_NO_3_S	0.3217764
86	Trans-4-hydroxy-L-proline	Amino acid derivatives	C_5_H_9_NO_3_	15,543.54
87	Glutamine	Amino acid derivatives	C_5_H_10_N_2_O_3_	3402.09
88	Aceglutamide	Amino acid derivatives	C_7_H_12_N_2_O_4_	2753.85
89	3-hydroxymethylglutaric acid	Amino acid derivatives	C_6_H_10_O_5_	1395.51
90	Methionine sulfoxide	Amino acid derivatives	C_5_H_11_NO_3_S	936.9
91	2-aminoisobutyric acid	Amino acid derivatives	C_4_H_9_NO_2_	569.7132
92	Kynurenic acid	Amino acid derivatives	C_10_H_7_NO_3_	538.8558
93	N-A-acetyl-L-arginine	Amino acid derivatives	C_8_H_16_N_4_O_3_	253.0788
94	Dimethylglycine	Amino acid derivatives	C_4_H_9_NO_2_	196.1832
95	N6-acetyl-L-lysine	Amino acid derivatives	C_8_H_16_N_2_O_3_	176.6814
96	L-cystathionine	Amino acid derivatives	C_7_H_14_N_2_O_4_S	137.0454
97	N-acetyl-L-tyrosine	Amino acid derivatives	C_11_H_13_NO_4_	130.8588
98	3-NmMethyl-L-histidine	Amino acid derivatives	C_7_H_11_N_3_O_2_	60.3822
99	S-(5-adenosy)-L-homocysteine	Amino acid derivatives	C_14_H_20_N_6_O_5_S	43.86558
100	3-hydroxyhippuric acid	Amino acid derivatives	C_9_H_9_NO_4_	38.94438
101	5-hydroxylysine	Amino acid derivatives	C_6_H_14_N_2_O_3_	37.97568
102	L-tyrosine methyl ester	Amino acid derivatives	C_10_H_13_NO_3_	9.99594
103	Xanthurenic acid	Amino acid derivatives	C_10_H_7_NO_4_	0.859944
104	Phe Met	Small peptide	C_14_H_20_N_2_O_3_S	31,754.34
105	Gamma-Glu-Met	Small peptide	C_10_H_18_N_2_O_5_S	8757.42
106	H-Glu(Leu-OH)-OH	Small peptide	C_11_H_20_N_2_O_5_	2768.154
107	L-alpha-aspartyl-L-phenylalanine	Small peptide	C_13_H_16_N_2_O_5_	1252.206
108	Gamma-glutamylalanine	Small peptide	C_8_H_14_N_2_O_5_	1129.818
109	Prolylhydroxyproline	Small peptide	C_10_H_16_N_2_O_4_	704.256
110	Glycyl-L-Proline	Small peptide	C_7_H_12_N_2_O_3_	647.298
111	Gamma-Glu-Phe	Small peptide	C_14_H_18_N_2_O_5_	179.1786
112	L-2-phenylglycine	Small peptide	C_8_H_9_NO_2_	120.2412
113	D-alanyl-D-alanine	Small peptide	C_6_H_12_N_2_O_3_	66.7956
114	Anserine	Small peptide	C_10_H_16_N_4_O_3_	59.87802
115	Leu-Ala	Small peptide	C_9_H_18_N_2_O_3_	57.22938
116	Phe Val	Small peptide	C_14_H_20_N_2_O_3_	1.918536
117	Biotin	Coenzymes and vitamins	C_10_H_16_N_2_O_3_S	10,261.5
118	Nadide	Coenzymes and vitamins	C_21_H_27_N_7_O_14_P_2_	9476.7
119	Pyridoxal hydrochloride	Coenzymes and vitamins	C_8_H_10_ClNO_3_	2699.274
120	Riboflavin sodium phosphate	Coenzymes and vitamins	C_17_H_20_N_4_NaO_9_P	389.1276
121	vitamin B1	Coenzymes and vitamins	C_12_H_17_ClN_4_OS	362.4606
122	Flavin mononucleotide	Coenzymes and vitamins	C_17_H_21_N_4_O_9_P	230.5356
123	Trigonelline	Coenzymes and vitamins	C_7_H_7_NO_2_	150.8532
124	Dethiobiotin	Coenzymes and vitamins	C_10_H_18_N_2_O_3_	142.0878
125	Pyridoxamine dichlorohydrate	Coenzymes and vitamins	C_8_H_14_C_l2_N_2_O_2_	54.82812
126	Pantothenol	Coenzymes and vitamins	C_9_H_19_NO_4_	49.37106
127	Niacinamide	Coenzymes and vitamins	C_6_H_6_N_2_O	28.4085
128	Acetyl-carnitine	CAR	C_9_H_17_NO_4_	7657.56
129	2-methylbutyroylcarnitine	CAR	C_12_H_23_NO_4_	7291.62
130	DL-carnitine	CAR	C_7_H_15_NO_3_	6279.66
131	Isovaleryl-carnitine	CAR	C_12_H_23_NO_4_	5110.812
132	Isobutyryl-L-carnitine	CAR	C_11_H_21_NO_4_	1932.33
133	L-palmitoylcarnitine	CAR	C_23_H_46_NO_4_	8.33856
134	Octanoyl-carnitine	CAR	C_15_H_29_NO_4_	4.018992
135	Myristoyl-L-carnitine	CAR	C_21_H_42_ClNO_4_	1.626294
136	Dodecanoylcarnitine	CAR	C_19_H_38_NO_4_	0.2799738
137	Decanoyl-carnitine	CAR	C_17_H_33_NO_4_	0.135042
138	Urea	Amines	CH_4_N_2_O	44,732.22
139	Trimethylamine N-oxide	Amines	C_3_H_9_NO	6001.5
140	Oleamide	Amines	C_18_H_35_NO	4558.662
141	Hexadecanamide	Amines	C_16_H_33_NO	946.986
142	5-methoxytryptamine	Amines	C_11_H_14_N_2_O	479.9064
143	3-methyl-1-butylamine	Amines	C_5_H_13_N	51.13974
144	Benzamide	Amines	C_7_H_7_NO	7.99776
145	N-cyclohexylformamide	Amines	C_7_H_13_NO	2.7777
146	1-monopalmitoylglycerol	FFA	C_19_H_38_O_4_	51,437.4
147	Apigenic acid	FFA	C_18_H_34_O_2_	8156.88
148	Transvaccenic acid (C18:1T)	FFA	C_18_H_34_O_2_	3299.142
149	Octadecanedioate (C18)	FFA	C_18_H_34_O_4_	338.8098
150	2-hydroxyhexadecanoic acid	FFA	C_16_H_32_O_3_	22.30626
151	Benzoic acid	Benzene and substituted derivatives	C_7_H_6_O_2_	10,863.42
152	Tyramine	Benzene and substituted derivatives	C_8_H_11_NO	625.092
153	2-phenylacetamide	Benzene and substituted derivatives	C_8_H_9_NO	25.30776
154	Phenylethylamine	Benzene and substituted derivatives	C_8_H_11_N	25.27692
155	4-hydroxybenzaldehyde	Benzene and substituted derivatives	C_7_H_6_O_2_	24.20568
156	Cholic acid	Bile acids	C_24_H_40_O_5_	256.8258
157	Taurocholic acid	Bile acids	C_26_H_45_NO_7_S	1.507548
158	Glycocholic acid	Bile acids	C_26_H_43_NO_6_	1.366428
159	Lumichrome	Heterocyclic compounds	C_12_H_10_N_4_O_2_	293.6082
160	2-aminobenzoic acid	Phenolic acids	C_7_H_7_NO_2_	267.9906
162	2-piperidone	Heterocyclic compounds	C_5_H_9_NO	247.3776
163	Biopterin	Pteridines and derivatives	C_9_H_11_N_5_O_3_	59.94504
164	4-hydroxybenzoic acid	Phenolic acids	C_7_H_6_O_3_	58.10418
165	Betaine aldehyde chloride	Others	C_5_H_12_ClNO	45.2907
166	Pyrrole-2-carboxylic acid	Heterocyclic compounds	C_5_H_5_NO_2_	20.29596
167	Dihydrotestosterone	Hormones and hormone-related compounds	C_19_H_30_O_2_	19.09512
168	Indole-3-carboxaldehyde	Indole and its derivatives	C_9_H_7_NO	18.5718
169	17a-Estradiol	Hormones and hormone-related compounds	C_18_H_24_O_2_	7.79946
170	Palmitoyl ethanolamide	LPE	C_18_H_37_NO_2_	6.8454
171	1-octadecylglycero-3-phosphocholine	Others	C_26_H_56_NO_6_P	5.111292
172	1-palmitoyl-sn-glycero-3-phosphocholine	LPC	C_24_H_50_NO_7_P	4.477656
173	Quinoline-2-carboxylic acid	Pteridines and derivatives	C_10_H_7_NO_2_	1.756626
174	Taurocholic acid	Bile acids	C_26_H_45_NO_7_S	1.507548
175	Glycocholic acid	Bile acids	C_26_H_43_NO_6_	1.366428
176	1-hexadecanoyl-sn-glycero-3-phosphoethanolamine	LPE	C_21_H_44_NO_7_P	0.737412
177	L-pipecolic Acid	Heterocyclic compounds	C_6_H_11_NO_2_	1895.538
178	Acetamide	Carboximidic acids	CH_3_CONH_2_	41,799.18
179	NADH	Dinucleotides	C_21_H_29_N_7_O_14_P_2_	22,004.58
180	Oleoylethanolamide	Fatty acyl	C_20_H_39_NO_2_	0.2143722
181	DL-mevalonic acid lactone	Esters	C_6_H_10_O_3_	1863.324
182	Indolelactic acid	Indole and its derivatives	C_11_H_11_NO_3_	3846.162
183	D-glucose 6-phosphate	Phosphate sugars	C_6_H_13_O_9_P	3526.254
184	Taurine	Sulfonic acids	C_2_H_7_NO_3_S	2789.094
185	1,5-anhydro-D-glucitol	Sugar alcohols	C_6_H_12_O_5_	1732.818
186	Tryptophol	Indole and its derivatives	C_10_H_11_NO	438.7266
187	2-picolinic acid	Pteridines and derivatives	C_6_H_5_NO_2_	805.35
188	Hypotaurine	Sulfonic acids	C_2_H_7_NO_2_S	652.476
189	Phosphorylethanolamine	LPE	C_2_H_8_NO_4_P	543.3204
190	dUMP	Nucleotide metabolomics	C_9_H_13_N_2_O_8_P	807.042
191	Glycerol 3-phosphate	Phosphoric acids	C_3_H_9_O_6_P	87,501.6
192	D-erythrose 4-phosphate	Phosphate sugars	C_4_H_9_O_7_P	75,813
193	Xyluose-5-phosphate	Phosphate sugars	C_5_H_11_O_8_P	14,043.66
194	Ethanolamine	Polyamines	C_2_H_7_NO	11,848.26
195	2-methylbutanoic acid	Fatty acids	C_5_H_10_O_2_	11,774.04
196	2-(formylamino)benzoic acid	Phenolic acids	C_8_H_7_NO_3_	11,033.76
197	Mannose	Sugars	C_6_H_12_O_6_	6619.68
198	2-amino-2-deoxymannose	Sugars	C_6_H_13_NO_5_	4629.444

**Figure A1:**
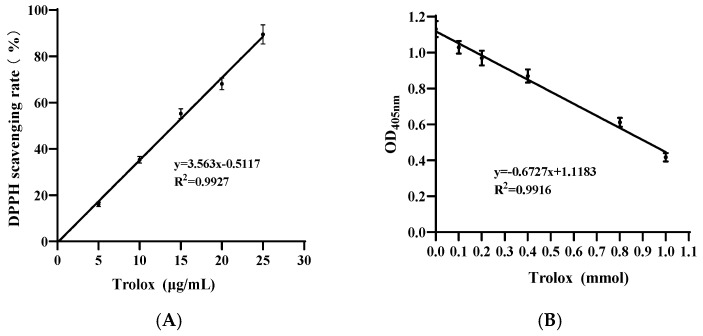
The standard curve. (**A**) DPPH scavenging rate. (**B**) total antioxidant capacity.

## Data Availability

Data collection for this project is ongoing; however, all datasets utilized in this research can be obtained by reaching out to the authors.

## Abbreviations

The following abbreviations are used in this manuscript:

UC	Ulcerative colitis
IBD	Inflammatory bowel disease
Pa JY062	Lacticaseibacillus paracasei JY062 postbiotic
ROS	Reactive oxygen species
Ctrl	Control
DSS	Dextran sulfate sodium
LP	Low-dose Pa JY062
MP	Medium-dose Pa JY062
HP	High-dose Pa JY062
T-SOD	Total superoxide dismutase
GSH-Px	Glutathione peroxidase
Th1/Th2	Helper T 1 cell/Helper T 2 cell
Th17/Treg	Helper T 17 cell/Regulatory T cells
ZO-1	Zona occludens 1
DAI	Disease activity index
SCFAs	Short-chain fatty acids
5-ASA	5-aminosalicylic acid
TNF-α	Tumor necrosis factor-α
IFN-γ	Interferon-γ
IL-2	Interleukin 2
IL-4	Interleukin 4
IL-10	Interleukin 10
IL-17A	Interleukin 17A
VCAM-1	Vascular cell adhesion molecule 1
ICAM-1	Intercellular cell adhesion molecule-1
LGG	Lacticaseibacillus rhamnosus GG
DPPH	1,1-diphenyl-2-picryl-hydrazyl radical
OD	Optical density
T-AOC	Total Antioxidant Capacity
DMEM	Dulbecco’s modification of Eagle’s medium
DCFH-DA	2′,7′-Dichlorodihydrofluorescein diacetate
PBS	Phosphate-buffer saline
H&E	Hematoxylin and eosin
AB-PAS	Alcian blue periodic acid–Schiff
IHC	Immunohistochemistry
RT-qPCR	Real-time quantitative polymerase chain reaction
GAPDH	Glyceraldehyde-3-phosphate dehydrogenase
HRP	Horseradish peroxidase
CXCL-1/3/5	C-X-C Motif Chemokine Ligand 1/3/5
MCP-1	Monocyte chemoattractant protein-1
TGF-β	Transforming growth factor-β
ELISA	Enzyme-linked immunosorbent assay
PCA	Principal component analysis
IECs	Intestinal epithelial cells

## Appendix A

### Appendix A.1. Evaluation of Oxidative Stress

#### Appendix A.1.1. Assay of Glutathione Peroxidase (GSH-PX)

Colon tissues were dissected into 5 mm^2^ fragments, flash-frozen in liquid nitrogen, and homogenized in ice-cold physiological saline (1:9 *w*/*v* ratio). After low-temperature centrifugation (3500 rpm, 10 min), the supernatant was collected. Protein concentrations were quantified via a bicinchoninic acid (BCA) assay kit.

For enzymatic assays, 0.2 mL of 1 mmol/L GSH and 0.2 mL homogenate were added to the enzyme group, while the non-enzyme control received only 0.2 mL GSH. Both tubes were preheated (37 °C, 5 min), followed by sequential addition of 0.1 mL Reagent 1 (37 °C incubation, 5 min) and 2 mL Reagent 2. Post-centrifugation (4000 rpm, 10 min), 1 mL of supernatant was transferred for chromogenic reaction. Blank (1.0 mL standard solvent), standard (1.0 mL 20 μmol/L GSH), and sample tubes (1.0 mL supernatant) were treated with Reagents 3 (1.0 mL), 4 (0.25 mL), and 5 (0.05 mL), incubated at 25 °C for 15 min, and quantified via OD412nm measurements.

GSH-Px activity was calculated as

Activity (U/mg protein) = (*A*_non-enzyme_ − *A*_enzyme_/(*A*_standard_ − *A*_blank_) × (*C*_standard_ × *N*)/(*T* × (*V*_sample_ × *C*_pr_))

Parameters:

*C*_standard_: GSH standard solution concentration (20 μmol/L) in chromogenic reaction

*N*: dilution factor (fixed at 5);

*T*: enzymatic reaction duration (5 min);

*V*_sample_: homogenate volume in reaction (0.2 mL);

*C*_pr_: homogenate protein concentration (mg protein/mL).

#### Appendix A.1.2. Assay of Superoxide Dismutase (SOD)

Colon tissues were processed identically to the GSH-Px assay. Assay tubes received sequential additions of 1.0 mL reagent 1, 0.05 mL sample, and 0.1 mL each of reagents 2–4, whereas control tubes substituted the sample with 0.05 mL distilled water. Samples were vortex-mixed thoroughly and incubated at 37 °C for 40 min. Post-incubation, 2 mL chromogenic developer was added to each tube with immediate mixing, followed by 10 min stabilization at 20–25 °C. Absorbance at 550 nm was measured against a distilled water blank.

Total SOD activity (U/mgprot) = (*OD*_control_ − *OD*_determination_)/(*OD*_control_) × (*V*_total_)/(50% × *V*_sample_ × *C*_pr_)

Parameters:

*V*_total_: total reaction volume (mL)

*V*_sample_: sample volume in reaction (mL)

*C*_pr_: homogenate protein concentration (mgpro/mL)

### Appendix A.2. 16S rRNA Analysis

#### Appendix A.2.1. Alpha Diversity

Alpha diversity is applied in analyzing complexity of species diversity for a sample through 6 indices, including observed-species, Chao1, Shannon, Simpson, ACE, good-coverage. All these indices in our samples were calculated with QIIME 2 (version 2024.5) and displayed with R software (Version 4.1.2).

Two indices were selected to identify community richness:

Chao—the Chao1 estimator (http://www.mothur.org/wiki/Chao (accessed on 25 July 2024));

ACE—the ACE estimator (http://www.mothur.org/wiki/Ace (accessed on 25 July 2024));

Two indices were used to identify community diversity:

Shannon—the Shannon index (http://www.mothur.org/wiki/Shannon (accessed on 25 July 2024));

Simpson—the Simpson index (http://www.mothur.org/wiki/Simpson (accessed on 25 July 2024));

One indice was used to characterized Sequencing depth:

Coverage—the Good’s coverage (http://www.mothur.org/wiki/Coverage (accessed on 25 July 2024)).

#### Appendix A.2.2. Beta Diversity

Beta diversity analysis was used to evaluate differences in samples in species complexity, Beta diversity on both weighted and unweighted unifrac were calculated by QIIME software. Cluster analysis was preceded by principal component analysis (PCA), which was applied to reduce the dimension of the original variables using the stats package and ggplot2 package in R software. Unweighted pair-group method with arithmetic means (UPGMA) clustering was performed as a type of hierarchical clustering method to interpret the distance matrix using average linkage and was conducted by QIIME software.

#### Appendix A.2.3. Analysis of the Relative Abundance

The relative abundance of gut microbes across experimental groups at the phylum, genus, and species levels were conducted on the Metware Cloud platform (https://cloud.metware.cn (accessed on 15 March 2024)) using R v4.1.2 and ggplot2 v3.3.5.

### Appendix A.3. Detection of Short-Chain Fatty Acids

#### Appendix A.3.1. Chemicals and Reagents

Methyl tert-butyl ether (MTBE) were purchased from CNW (CNW Technologies, Düsseldorf, Germany). MilliQ water (Millipore, PA, USA) was used in all experiments. All of the standards were purchased from CNW (CNW, Beijing, China) or aladdin (Shanghai, China). The stock solutions of standards were prepared at the concentration of 1 mg/mL in MTBE. All stock solutions were stored at −20 °C. The stock solutions were diluted with MTBE to working solutions before analysis.

#### Appendix A.3.2. Sample Preparation and Extraction

A total of 20 mg of fecal sample were accurately weighed and placed in a 2 mL EP tube. 1 mL of phosphoric acid (0.5% *v*/*v*) solution and a small steel ball were added to the EP tube. The samples were ground uniformly, then vortexed for 10 min and ultrasonicated for 5 min. 100 μL of supernatant was moved into 1.5 mL centrifugal tube after the mixture was centrifuged with a speed of 12,000 r/min for 10 min at 4 °C. 500 μL of MTBE (containing internal standard) solution was added to the centrifugal tube and the mixture was vortexed for 3 min followed by ultrasonicating for 5 min. After that, the mixture was centrifuged with a speed of 12,000 r/min for 10 min at 4 °C. The supernatant was collected and used for GC-MS/MS analysis.

#### Appendix A.3.3. GC–MS Analysis

An Agilent 7890B gas chromatograph coupled to a 7000D mass spectrometer with a DB-FFAP column (30 m length × 0.25 mm i.d. × 0.25 μm film thickness, J&W Scientific, CA, USA) was employed for GC-MS/MS analysis of SCFAs. Helium was used as carrier gas, at a flow rate of 1.2 mL/min. Injection was made in the split mode with a split ratio 5:1 and the injection volume was 1 μL. The oven temperature was held at 50 °C for 1 min, raised to 220 °C at a rate of 18 °C/min and held for 5 min. All samples were analyzed in multiple reaction monitoring mode. The injector inlet and transfer line temperature were 250 °C and 230 °C, respectively.

**Table A1:** Subsequent operations of total antioxidant capacity (T-AOC).

Title 1	Blank Well	Standard Well	Test Well
Sterile water (μL)	10		
0.1, 0.2, 0.4, 0.8, and 1.0 mM Trolox (μL)		10	
Samples (μL)			10
Reagent IV application solution (μL)	20	20	20
ABTS working solution (μL)	170	170	170

**Table A2:** Operation steps of DPPH radical scavenging activity in EP tube.

Title 1	Control Tube	Sample Tube	Blank Tube
Sample (μL)	400	400	
80% methanol (μL)	600		400
Working solution (μL)		600	600

**Table A3:** RNA concentration.

Samples	RNA Concentration (ng/μL)
Ctrl 1	875
Ctrl 2	937
Ctrl 3	911
LPS 1	1062
LPS 2	1369
LPS 3	1136
LP 1	854
LP 2	761
LP3	1329
MP 1	1291
MP 2	1365
MP 3	1555
HP 1	955
HP 2	1029
HP 3	1422
ASA-1	852
ASA-2	1036
ASA-3	1112

**Table A4:** RT-qPCR primers.

Primer Name	Forward Primer (5′-3′)	Reverse Primer (5′-3′)
*Zo-1*	TGAGGCAGCTCACATAATGC	GGTCTCTGCTGGCTTGTTTC
*Occludin*	AAAGGGCATTGCTCATCCTGA	ACAATGGCAATGGCAATTCATC
*Claudin-1*	CCAGTCAATGCCAGGTACGAAT	GGCCTTGGTGTTGGGTAAGA
*E-cadherin*	CCCAAACGTAACGAGGGTATC	GGCAGCTTGAAGTGGTAGAAGT
*CXCL-1*	TGCACCCAAACCGAAGTCAT	ACTTGGGGACACCTTTTAGCAT
*MCP-1*	CAGGTCCCTGTCATGCTTCT	CCCATTCCTTCTTGGGGTCA
*CXCL-3*	TGAGGCAGTATTCCTTGGCTG	ACCGGCATGACCTTGTTTGT
*CXCL-5*	TCCTCAGTCATAGCCGCAAC	TAGCTTTCTTTTTGTCACTGCCC
*IL-10*	GGGTTGCCAAGCCTTGTCTGAG	CCTTGATGTCTGGGTCTTGGTTCTC
*IL-17A*	GTTAGGGTGCTTTAGGTCC	TAACAATGAGTTTCTGTACG
*TGF-β*	TACAGCAACAATTCCTGGCGATACC	CTCAACCACTGCCGCACAACTC
*GAPDH*	GAGAAGGCTGGGGCTCATTT	TAAGCAGTTGGTGGTGCAGG

**Table A5:** Postbiotic components of PaJY062 with active function.

	Compounds	Function	References
1	Allantoin	Antioxidant; anti-inflammatory	[70]
2	Trehalose	Antioxidant; anti-inflammatory; repair the intestinal barrier	[71]
3	Histidine	Antioxidant; anti-inflammatory; repair the intestinal barrier	[26,72]
4	L-Norvaline	Anti-inflammatory	[73]
5	Phenylalanine	Antioxidant; anti-inflammatory; repair the intestinal barrier	[74]
6	Glycine	Antioxidant; anti-inflammatory	[75,76]
7	Tryptophan	Anti-inflammatory	[77]
8	Betaine	Antioxidant; anti-inflammatory; repair the intestinal barrier	[78,79]
9	Citric acid	Antioxidant	[80]
10	3-phenyllactic acid	Antioxidant	[81]
11	Threonine	Antioxidant; anti-inflammatory; repair the intestinal barrier	[82]
12	Benzoic acid	Repair the intestinal barrier	[83]
13	Biotin	Anti-inflammatory; repair the intestinal barrier	[84]
14	Serine	Anti-inflammatory; repair the intestinal barrier	[85]
15	Mannose	Antioxidant; anti-inflammatory; repair the intestinal barrier; modulate the gut microbiota	[86]
16	Oleamide	Anti-inflammatory	[87]
17	Glutamine	Anti-inflammatory; repair the intestinal barrier; modulate the gut microbiota	[88]
18	Taurine	Antioxidant; anti-inflammatory; repair the intestinal barrier	[89]
19	Salicylic acid	Anti-inflammatory; repair the intestinal barrier	[90]
20	Hexadecanamide	Anti-inflammatory	[91]
21	Adenosine	Antioxidant; anti-inflammatory; repair the intestinal barrier	[92]
22	Quinic acid	Antioxidant; anti-inflammatory; repair the intestinal barrier	[93]
23	Hypotaurine	Antioxidant	[94]
24	Stachydrine hydrochloride	Anti-inflammatory	[95]
25	Dimethylglycine	Anti-inflammatory; repair the intestinal barrier; modulate the gut microbiota	[96]
26	Trigonelline	Anti-inflammatory	[97]
27	Anserine	Antioxidant	[98]
28	Niacinamide	Antioxidant; anti-inflammatory	[99]
29	4-hydroxybenzaldehyde	Antioxidant	[100]
30	17α-estradiol	Anti-inflammatory	[101]
31	Palmitoyl ethanolamide	Anti-inflammatory	[102]
